# Radiomic Model Associated with Tumor Microenvironment Predicts Immunotherapy Response and Prognosis in Patients with Locoregionally Advanced Nasopharyngeal Carcinoma

**DOI:** 10.34133/research.0749

**Published:** 2025-06-24

**Authors:** Jie Sun, Xuewei Wu, Xiao Zhang, Weiyuan Huang, Xi Zhong, Xueyan Li, Kaiming Xue, Shuyi Liu, Xianjie Chen, Wenzhu Li, Xin Liu, Hui Shen, Jingjing You, Wenle He, Zhe Jin, Lijuan Yu, Yuange Li, Shuixing Zhang, Bin Zhang

**Affiliations:** ^1^ Department of Radiology, The First Affiliated Hospital of Jinan University, Guangzhou, Guangdong, China.; ^2^ Medical AI Lab, The First Hospital of Hebei Medical University, Hebei Medical University, Shijiazhuang, Hebei, China.; ^3^ Hebei Provincial Engineering Research Center for AI-Based Cancer Treatment Decision-Making, The First Hospital of Hebei Medical University, Hebei Medical University, Shijiazhuang, Hebei, China.; ^4^ Department of Oncology, The First Hospital of Hebei Medical University, Hebei Medical University, Shijiazhuang, Hebei, China.; ^5^ Department of Radiology, Hainan Affiliated Hospital of Hainan Medical University (Hainan General Hospital), Haikou, Hainan, China.; ^6^ Department of Medical Imaging, Guangzhou Institute of Cancer Research, the Affiliated Cancer Hospital, Guangzhou Medical University, Guangzhou, Guangdong, China.; ^7^ Department of Radiology, Hainan Cancer Hospital, Haikou, Hainan, China.; ^8^ Department of Radiology, The Third Bethune Hospital of Jilin University, Changchun, Jilin, China.; ^9^ Department of Radiology, Guangzhou Women and Children’s Medical Center, Guangzhou Medical University, Guangzhou, Guangdong, China.; ^10^ Department of Radiology, The Affiliated Panyu Central Hospital, Guangzhou Medical University, Guangzhou, Guangdong, China.; ^11^ Department of Radiology, Affiliated Hospital of Guangdong Medical University, Zhanjiang, Guangdong, China.

## Abstract

**Background:** No robust biomarkers have been identified to predict the efficacy of programmed cell death protein 1 (PD-1) inhibitors in patients with locoregionally advanced nasopharyngeal carcinoma (LANPC). We aimed to develop radiomic models using pre-immunotherapy MRI to predict the response to PD-1 inhibitors and the patient prognosis. **Methods:** This study included 246 LANPC patients (training cohort, *n* = 117; external test cohort, *n* = 129) from 10 centers. The best-performing machine learning classifier was employed to create the radiomic models. A combined model was constructed by integrating clinical and radiomic data. A radiomic interpretability study was performed with whole slide images (WSIs) stained with hematoxylin and eosin (H&E) and immunohistochemistry (IHC). A total of 150 patient-level nuclear morphological features (NMFs) and 12 cell spatial distribution features (CSDFs) were extracted from WSIs. The correlation between the radiomic and pathological features was assessed using Spearman correlation analysis. **Results:** The radiomic model outperformed the clinical and combined models in predicting treatment response (area under the curve: 0.760 vs. 0.559 vs. 0.652). For overall survival estimation, the combined model performed comparably to the radiomic model but outperformed the clinical model (concordance index: 0.858 vs. 0.812 vs. 0.664). Six treatment response-related radiomic features correlated with 50 H&E-derived (146 pairs, |*r*|= 0.31 to 0.46) and 2 to 26 IHC-derived NMF, particularly for CD45RO (69 pairs, |*r*|= 0.31 to 0.48), CD8 (84, |*r*|= 0.30 to 0.59), PD-L1 (73, |*r*|= 0.32 to 0.48), and CD163 (53, |*r*| = 0.32 to 0.59). Eight prognostic radiomic features correlated with 11 H&E-derived (16 pairs, |*r*|= 0.48 to 0.61) and 2 to 31 IHC-derived NMF, particularly for PD-L1 (80 pairs, |*r*|= 0.44 to 0.64), CD45RO (65, |*r*|= 0.42 to 0.67), CD19 (35, |*r*|= 0.44 to 0.58), CD66b (61, |*r*| = 0.42 to 0.67), and FOXP3 (21, |*r*| = 0.41 to 0.71). In contrast, fewer CSDFs exhibited correlations with specific radiomic features. **Conclusion:** The radiomic model and combined model are feasible in predicting immunotherapy response and outcomes in LANPC patients. The radiology–pathology correlation suggests a potential biological basis for the predictive models.

## Introduction

Nasopharyngeal carcinoma (NPC), a highly invasive malignancy, is known for its propensity for local invasion and early onset of distant metastasis. At initial diagnosis, approximately 75% of patients present with locoregionally advanced NPC (LANPC) [1]. Concurrent chemoradiotherapy (CRT) has long been the cornerstone of curative treatment for LANPC. However, a growing body of evidence supports the integration of systemic therapy, including induction, concurrent, and adjuvant chemotherapy, to enhance disease control and survival. Notably, induction chemotherapy, particularly gemcitabine plus cisplatin, has shown substantial benefit in phase III trials, improving progression-free survival (PFS) and overall survival (OS) when administered prior to CRT [2]. Additionally, the ESMO-EURACAN Clinical Practice Guidelines recommend the use of adjuvant capecitabine in high-risk patients following CRT [3]. These advancements highlight a shift toward risk-adapted, personalized treatment strategies, particularly in patients with high Epstein–Barr virus (EBV) DNA levels or other unfavorable prognostic features.

Immunotherapy, particularly programmed cell death protein 1 (PD-1) inhibitors, has shown impressive results across various cancer types and considerably improved the efficacy of tumor therapy [4]. NPC is characterized by high programmed death-ligand 1 (PD-L1) expression and abundant tumor-infiltrating lymphocytes, providing a strong biological rationale for incorporating immunotherapy in the treatment of this disease [5–7]. Currently, several phase III trials have shown that the addition of PD-1 inhibitors to gemcitabine-cisplatin as a first-line treatment significantly prolongs PFS in patients with recurrent or metastatic NPC (R/M NPC). In LANPC, the efficacy of PD-1 inhibitors plus radiochemotherapy as a novel curative approach is actively being explored. Some phase II trials have confirmed favorable response rates and/or survival outcomes with PD-1 inhibitors in LANPC, reporting an objective response rate of 88.9% to 94.4% and 2-year PFS of 69.6% to 91.8% [8–13]. A recent large-scale phase III trial found that adding PD-1 inhibitors in treating patients with high-risk LANPC [14] resulted in a 40% decrease in recurrence and a 43% reduction in distant metastasis, markedly prolonging patient survival [1]. However, given the multitude of immune evasion strategies employed by tumors, only approximately 20% to 30% of patients benefit from immunotherapy [15]. Additionally, it can also cause immune-related adverse events that may affect multiple organs and can appear long after treatment ends, sometimes leading to treatment-related mortality [16,17]. Therefore, reliable biomarkers capable of predicting immunotherapy response are clinically imperative.

The current predictive capability of biomarkers, such as PD-L1 expression [18], EBV level [8], Ki67+ proliferating regulatory T cells (Tregs) [19], and tumor mutational burden (TMB) [20], has shown limited and unsatisfactory accuracy in forecasting the response to immunotherapy in NPC [14,19,21,22]. Hence, it is crucial to explore innovative methods for more accurate and noninvasive prediction of their efficacy. Radiomic analysis, as an integrated approach, represents a noninvasive method for this purpose. Radiomics refers to the extraction and analysis of quantitative features from medical images, enabling the capture of cellular and molecular properties of tissues [23,24]. Machine learning techniques facilitate high-throughput extraction of quantitative imaging features and further evaluation of the heterogeneity of tumor microenvironment (TME) [23]. The noninvasive and reliable property of radiomics provides an innovative approach to predicting response to immunotherapy [24]. Radiomic biomarkers have recently achieved success in assisting diagnosis and prognostic assessment, with studies highlighting the potential of radiomics in predicting the response to immune checkpoint inhibitor (ICI) treatment in lung cancer, breast cancer, and melanoma [25–27]. However, previous studies on imaging biomarkers aimed at predicting the efficacy of combined immunotherapy approaches for LANPC have small sample sizes and lack external validation cohorts as well as biointerpretability [28,29].

In the current study, we aimed to identify noninvasive radiomic biomarkers by extracting features from baseline MRI images of LANPC and to further investigate their ability to predict immunotherapy response and patient prognosis. The predictive accuracy of the radiomic models was validated against clinical models and tested for robustness using an independent test cohort. Furthermore, we conducted an extraction of pathological features from hematoxylin and eosin (H&E) and immunohistochemistry (IHC)-stained whole slide images (WSIs) and explored the correlations between the predictive radiomic and pathological features.

## Results

### Clinical characteristics of patients

A total of 246 eligible patients were included in this retrospective study. Detailed demographic and clinical characteristics are summarized in Table 1. In the training cohort, responses were as follows: 48 patients with immune partial response (iPR), 39 with immune complete response (iCR), 3 with immune confirmed progressive disease (iCPD), and 27 with immune stable disease (iSD), resulting in a 74% (87/117) response rate. The external test cohort showed 85 patients with iPR, 13 with iCR, 4 with iCPD, and 27 with iSD, with a 76% (98/129) response rate. Baseline characteristics did not differ significantly between responders and non-responders, except for white blood cell (WBC) count, lymphocytes in the training cohort, which were statistically significant (*P* < 0.05).

**Table 1.** Demographics and clinicopathologic characteristics of the training and external test cohorts. Data are presented as number (percentage) and mean ± standard deviation (SD). All variables were collected before immunotherapy rather than the initial diagnosis.

Variables	Entire cohort (*N* = 246)	Training cohort (*n* = 117)			External test cohort (*n* = 129)		
		Responders (*n* = 87)	Non-responders (*n* = 30)	*P* value	Responders (*n* = 98)	Non-responders (*n* = 31)	*P* value
**Age (years)**	49.7 ± 12.6	51.2 ± 11.3	54.7 ± 12.7	0.160	48.5 (38.0–58.3)	42.0 (35.0–58.0)	0.281
**Sex**				0.091			0.263
Male	172 (69.9)	61 (70.1)	26 (86.7)		62 (63.3)	23 (74.2)	
Female	74 (30.1)	26 (29.9)	4 (13.3)		36 (36.7)	8 (25.8)	
**T stage**				0.139			0.871
T1	11 (4.5)	4 (4.8)	2 (7.4)		4 (4.1)	1 (3.2)	
T2	37 (15.0)	15 (17.2)	3 (10.0)		16 (16.3)	3 (9.7)	
T3	114 (46.3)	38 (43.7)	8 (26.7)		51 (52.0)	17 (54.8)	
T4	84 (34.1)	30 (34.5)	17 (56.7)		27 (27.6)	10 (32.3)	
**N stage**				0.091			0.644
N0	19 (7.7)	5 (5.7)	5 (16.7)		8 (8.2)	1 (3.2)	
N1	54 (22.0)	18 (20.7)	10 (33.3)		18 (18.4)	8 (25.8)	
N2	95 (38.6)	39 (44.8)	10 (33.3)		34 (34.7)	12 (38.7)	
N3	78 (31.7)	25 (28.7)	5 (16.7)		38 (38.8)	10 (32.3)	
**Clinical stage**				0.840			0.566
1	1 (0.4)	0	0		1 (1.0)	0	
2	14 (5.7)	7 (8.0)	1 (3.3)		6 (6.1)	0	
3	89 (36.2)	31 (35.6)	11 (36.7)		34 (34.7)	13 (41.9)	
4a	142 (57.7)	49 (56.3)	18 (60.0)		57 (58.1)	18 (58.1)	
**EBV DNA**				0.398			0.744
Negative	131 (53.3)	40 (46.0)	17 (56.7)		57 (58.2)	17 (54.8)	
Positive	115 (46.7)	47 (54.0)	13 (43.3)		41 (41.8)	14 (45.2)	
**Neutrophils** (10^9^/l)	5.1 ± 2.8	4.2 (3.3–5.5)	3.2 (2.8–5.0)	0.055	3.5 (2.4–5.1)	3.7 (3.0–6.8)	0.058
**Lymphocytes** (10^9^/l)	2.4 ± 2.3	1.4 (1.1–1.9)	1.2 (0.8–1.4)	0.045	1.3 (0.8–1.9)	1.3 (0.8–1.7)	0.921
**NLR**	3.5 ± 4.0	3.3 (2.1–4.6)	3.6 (1.9–4.9)	0.822	2.8 (1.8–4.5)	3.6 (2.6–4.8)	0.078
**WBC** (10^9^/l)	4.7 ± 3.0	6.7 ± 2.0	5.7 ± 1.7	0.016	6.1 ± 3.0	7.4 ± 3.1	0.050
**Hemoglobin** (g/l)	128.3 ± 20.3	129.3 ± 17.4	124.3 ± 18.8	0.186	129.08 ± 22.2	125.52 ± 23.7	0.621
**Platelet counts** (10^9^/l)	267.3 ± 90.4	262.0 (227.0–316.0)	235.0 (174.8–300.5)	0.184	254.8 (205.2–303.7)	264.0 (198.0–314.0)	0.493
**Immunotherapy regimens**				0.465			0.196
Neoadjuvant	149 (60.6)	56 (64.4)	17 (56.7)		57 (58.2)	19 (61.3)	
Concurrent	5 (2.0)	1 (1.1)	1 (3.3)		1 (1.0)	2 (6.5)	
Adjuvant	92 (37.4)	30 (34.5)	12 (40.0)		40 (40.8)	10 (32.3)	

NLR, neutrophil-to-lymphocyte ratio; WBC, white blood cell

### Predictive performance of the models for immunotherapy response

**Fig. 1.**
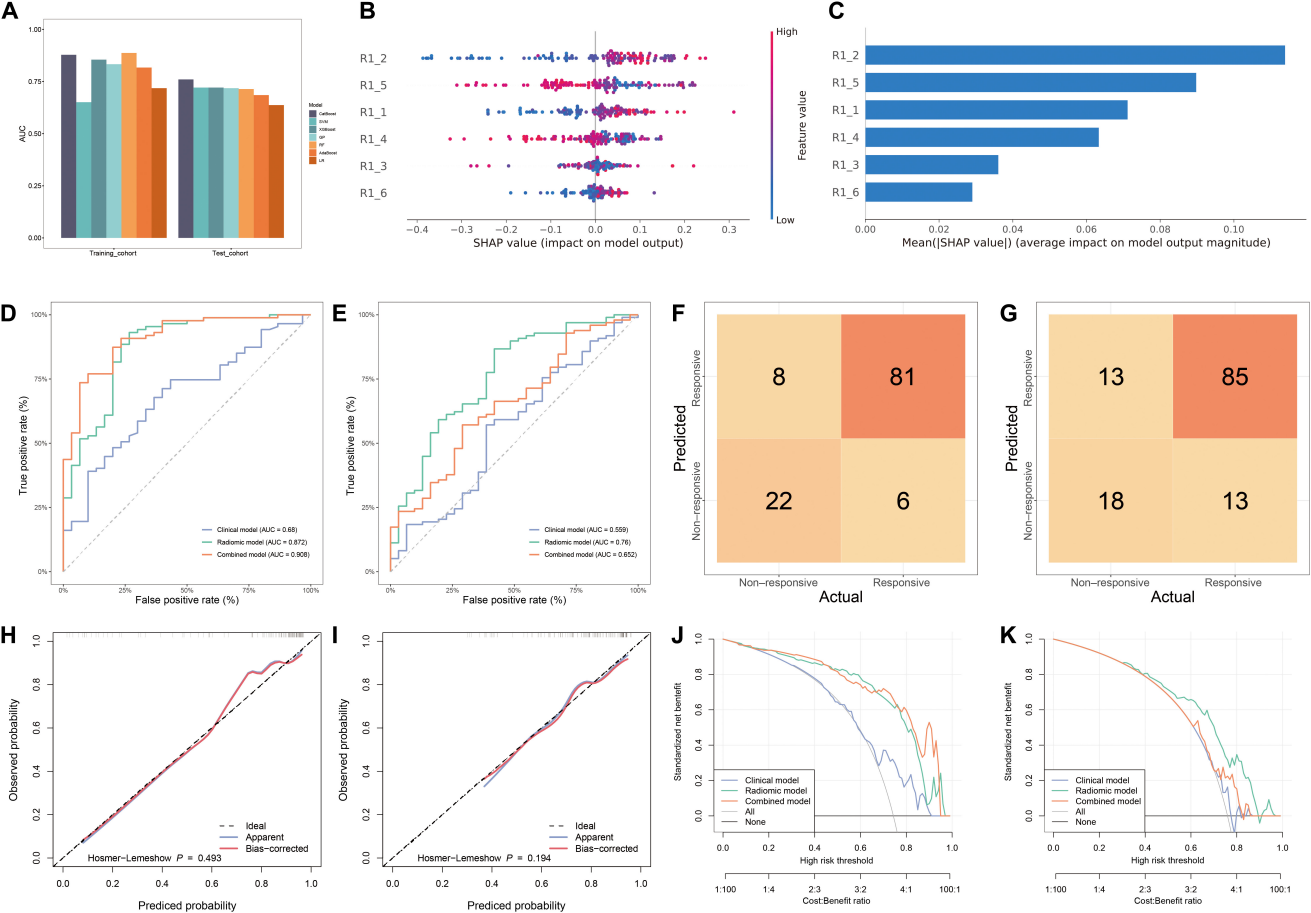
Performance of the 3 developed models for predicting treatment response. The histogram displays the AUC values for 7 radiomics-based machine learning algorithms in both training and external test cohorts (A). The SHAP beeswarm plot illustrates the importance of radiomic features, with the *x*-axis representing each feature’s impact on model prediction according to its SHAP value; the absolute value indicates the magnitude of the impact, while positive and negative values show increases and decreases in predicted probability (B). A bar graph ranks variables by their importance to the model, based on the mean of the absolute values of all Shapley values for 6 radiomic features (C). The ROC curves for the clinical model, radiomic model, and combined model are presented for both training (D) and external test cohorts (E), with the radiomic model demonstrating higher AUC values at most time points compared to the clinical and combined models. The confusion matrix of radiomic model using the training (F) and external test cohorts (G). Calibration plots display the apparent calibration curve and the bias-corrected calibration curve after bootstrapping for both the training and external test cohorts (H and I). Decision curve analysis for the combined model (orange), radiomics model (green), and clinical model (blue) in the training cohort (J) and external test cohort (K); the *y*-axis indicates the net benefit; the *x*-axis indicates threshold probability. The gray line represents the assumption that all patients were responders. The black line represents the hypothesis that no patients were responders. AUC, area under the curve; ROC, receiver operating characteristic; SHAP, Shapley Additive exPlanations; R1_1, T1C_wavelet-HHL_glcm_Imc2; R1_2, T1C_wavelet-HHL_glcm_MaximumProbability; R1_3, T2_ log-sigma-5-0-mm-3D_glcm_Autocorrelation; R1_4, T2_wavelet-LHL_firstorder_Median; R1_5, T2_wavelet-LHL_glcm_Autocorrelation; R1_6, T2_wavelet-LHL_glcm_ClusterShade.

The stability of lesion segmentation and the robustness of features across different segmentations are satisfactory (Table S1). After reducing redundancy through Pearson correlation analysis (PCC) analysis, 1,409 features remained (T1-weighted imaging [T1WI], *n* = 441; T2-weighted imaging [T2WI], *n* = 463; contrast-enhanced T1WI [CET1WI], *n* = 505). Using the analysis of variance (ANOVA) and machine learning methods, the results indicated that the optimal model could be constructed with a CatBoost (Categorical Boosting) classifier (Fig. 1A). The radiomic model comprised 6 features: 2 from CET1WI and 4 from T2WI (Table S2). Subsequently, these radiomic features were used to construct a radiomic model for predicting response to immunotherapy. The SHapley Additive exPlanations (SHAP) beeswarm plot illustrates the relative importance of radiomic features. In this plot, the *x*-axis represents each feature’s impact on model prediction according to its SHAP value, with the absolute value indicating the magnitude of the impact and positive/negative values showing increases/decreases in the predicted probability. A bar graph ranks variables by their contribution to the model, based on the mean of the absolute values of all Shapley values for the 6 radiomic features (Fig. 1B and C). The area under the curve (AUC) values of the radiomic model were 0.872 (95% confidence interval [CI]: 0.840 to 0.904) in the training cohort and 0.760 (95% CI: 0.716 to 0.805) in the external test cohort (Table 2 and Fig. 1D and E).

**Table 2.** Performance of the models for predicting immunotherapy response

		AUC (95% CI)	*P* value	Sensitivity (%)	Specificity (%)	Accuracy (%)
**Training cohort**	Clinical model	0.680 (0.637–0.723)	<0.001	72.4	56.7	68.4
	Radiomic model	0.872 (0.840–0.904)	<0.001	88.5	73.3	84.6
	Combined model	0.908 (0.885–0.932)	Ref.	87.4	76.7	87.2
**External test cohort**	Clinical model	0.559 (0.506–0.612)	0.044	68.4	35.5	60.5
	Radiomic model	0.760 (0.716–0.805)	<0.001	77.6	58.1	72.9
	Combined model	0.652 (0.604–0.700)	Ref.	45.9	74.2	52.7

Clinical variables with statistical differences (both *P* < 0.05) in univariate and multivariate logistic regression analyses were employed to construct the clinical model. Only N stage (odds ratio [OR], 1.772; 95% CI:1.115 to 2.889) and WBC (OR 1.368; 95% CI:1.073 to 1.798) were included in the clinical model (Table S3), with AUC values of 0.680 (95% CI: 0.637 to 0.723) in the training cohort and 0.559 (95% CI: 0.506 to 0.612) in the external test cohort (Table 2). The combined model integrating N stage, WBC, and Radscore achieved AUC values of 0.908 (95% CI: 0.885 to 0.932) and 0.652 (95% CI: 0.604 to 0.700) in the training and testing cohorts, respectively (Table 2). The accuracy of the radiomic model in the training and the testing cohorts was 84.6% and 72.9%, respectively (Fig. 1F and G). The radiomic model AUC value was higher than the AUC value of 0.576 (95% CI: 0.386 to 0.744) of the single model of Combined Positive Score (CPS) of PD-L1 in the external validation cohort. The calibration curve (Fig. 1H and I) showed that the probability of immunotherapy response predicted by the radiomic model closely aligned with the actual probabilities, with Hosmer-Lemeshow *P* values of 0.493 and 0.194, respectively. The decision curve analysis (DCA) revealed that the radiomic model provided a superior net benefit compared to the clinical and radiomic models (Fig. 1J and K).

### Predictive performance of the models for prognosis

**Fig. 2.**
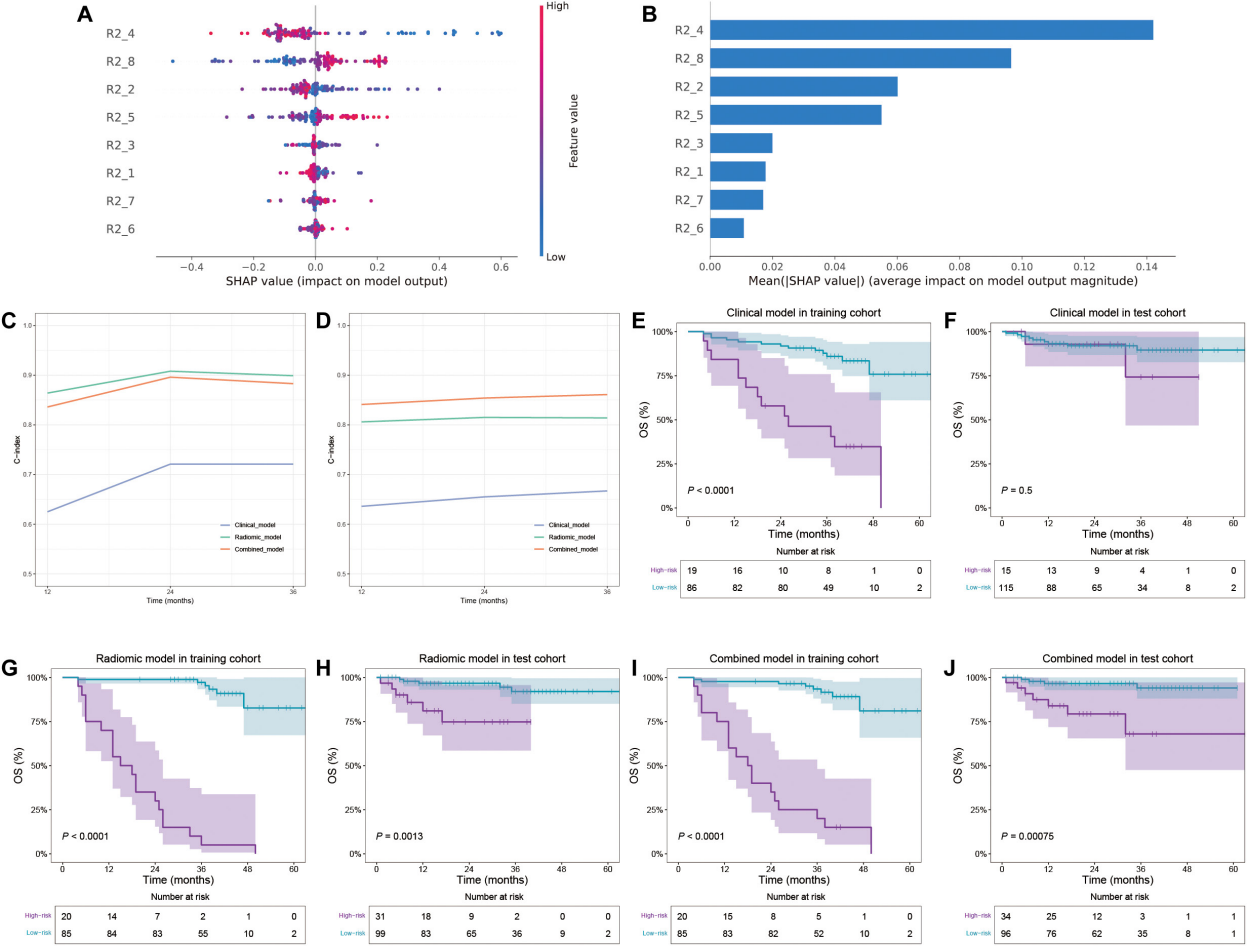
Performance of the 3 developed models for predicting prognosis. The SHAP beeswarm plot shows the positive or negative effects of each feature on the prediction probability through red and blue colors (A). A bar graph ranks variables by their importance to the model, based on the mean of the absolute values of all Shapley values for 8 radiomic features (B). Time-dependent concordance index (C-index) of 1-year, 2-year, and 3-year survival intervals, reflecting prediction performance at various time points. The top-performing model (combined model) is compared with the radiomic model and clinical model in both training (C) and external test cohorts (D). The Kaplan–Meier curves illustrate OS probabilities for patients stratified into low- and high-risk groups based on 3 models: the clinical model across the training (E) and external test cohorts (F), the radiomic model across the training (G) and external test cohorts (H), and the combined model across the training (I) and external test cohorts (J). OS, overall survival; AUC, area under the curve; SHAP, Shapley Additive exPlanations; R2_1, T1_wavelet-HLL_glrlm_ShortRunHighGrayLevelEmphasis; R2_2, T1_wavelet-HLL_gldm_LargeDependenceHighGrayLevelEmphasis; R2_3, T1_wavelet-HLH_glrlm_RunEntropy; R2_4, T1_wavelet-HLH_gldm_LargeDependenceLowGrayLevelEmphasis; R2_5, T1C_wavelet-HLL_glcm_Imc1; R2_6, T1C_wavelet-HLH_glcm_DifferenceEntropy; R2_7, T1C_wavelet-HLH_glrlm_RunVariance; R2_8, T2_wavelet-HLH_glcm_ClusterProminence.

After excluding patients with follow-up periods of less than 2 years, the training cohort comprised 105 patients, while the external test cohort included 130 patients. Following the removal of redundant features through PCC analysis, 1,400 features remained (T1WI, *n* = 440; CET1WI, *n* = 496; T2WI, *n* = 464). The ANOVA method combined with the Adaboost classifier identified an optimal model consisting of 8 features: 4 from T1WI, 3 from CET1WI, and 1 from T2WI (Table S4), which led to the derivation of the OS-Radscore. The SHAP beeswarm plot illustrates the relative importance of radiomic features (Fig. 2A and B). This radiomic model achieved the concordance index (C-index) of 0.860 (95% CI: 0.844 to 0.876) in the training cohort and 0.812 (95% CI: 0.783 to 0.841) in the external test cohort.

Following Cox analyses of clinical variables, only age, N stage, and hemoglobin emerged as independent risk factors (Table S5) and were finally included in the clinical model. This model achieved a C-index of 0.723 (95% CI: 0.688 to 0.758) in the training cohort and 0.664 (95% CI: 0.621 to 0.707) in the external test cohort (Table S6). Additionally, we integrated clinical features into the radiomic model to develop a combined model aimed at improving prediction performance. Stepwise multivariate Cox analysis with the clinical indicators age, N stage, and hemoglobin and the OS-Radscore showed that age, N stage, and OS-Radscore were retained in the final model. The inclusion of age, N stage, and OS-Radscore improved the C-index compared to the radiomics model alone, which achieved a C-index of 0.850 (95% CI: 0.821 to 0.879) in the training cohort and 0.858 (95% CI: 0.842 to 0.874) in the external test cohort (Table S6). All of the above model C-indices were higher than the C-index of 0.580 (95% CI: 0.309 to 0.858) of the single model of CPS scores of PD-L1 in the external validation cohort.

Meanwhile, we evaluated the predictive accuracy of the predictive models across 1-year, 2-year, and 3-year survival intervals. The combined model demonstrated superior performance in comparison to both the clinical and radiomic models, achieving higher C-indices in both the training (0.883, 95% CI: 0.822 to 0.940) and test cohorts (0.861,95% CI: 0.822 to 0.940), particularly for 3-year OS (Table S7). These findings suggest that the combined model exhibits enhanced predictive capability for survival outcomes in LANPC.

The combined model could divide patients into high- and low-risk subgroups with distinct survival probabilities using a threshold of 0.55. The Kaplan–Meier analysis displayed that the high-risk subgroup identified by the combined model had significantly shorter OS compared to the low-risk subgroup (all *P* < 0.05) (Fig. 2I and J).

The reporting and methodological quality of our study was evaluated by the METhodological RadiomICs Score (METRICS) tool (https://metricsscore.github.io/metrics/METRICS.html), which includes 30 items across 9 categories. The total METRICS score of our study was 89.4%, suggesting excellent quality (Fig. S1).

### Radiopathomic correlation analysis: Interpretability of the radiomic model

**Fig. 3.**
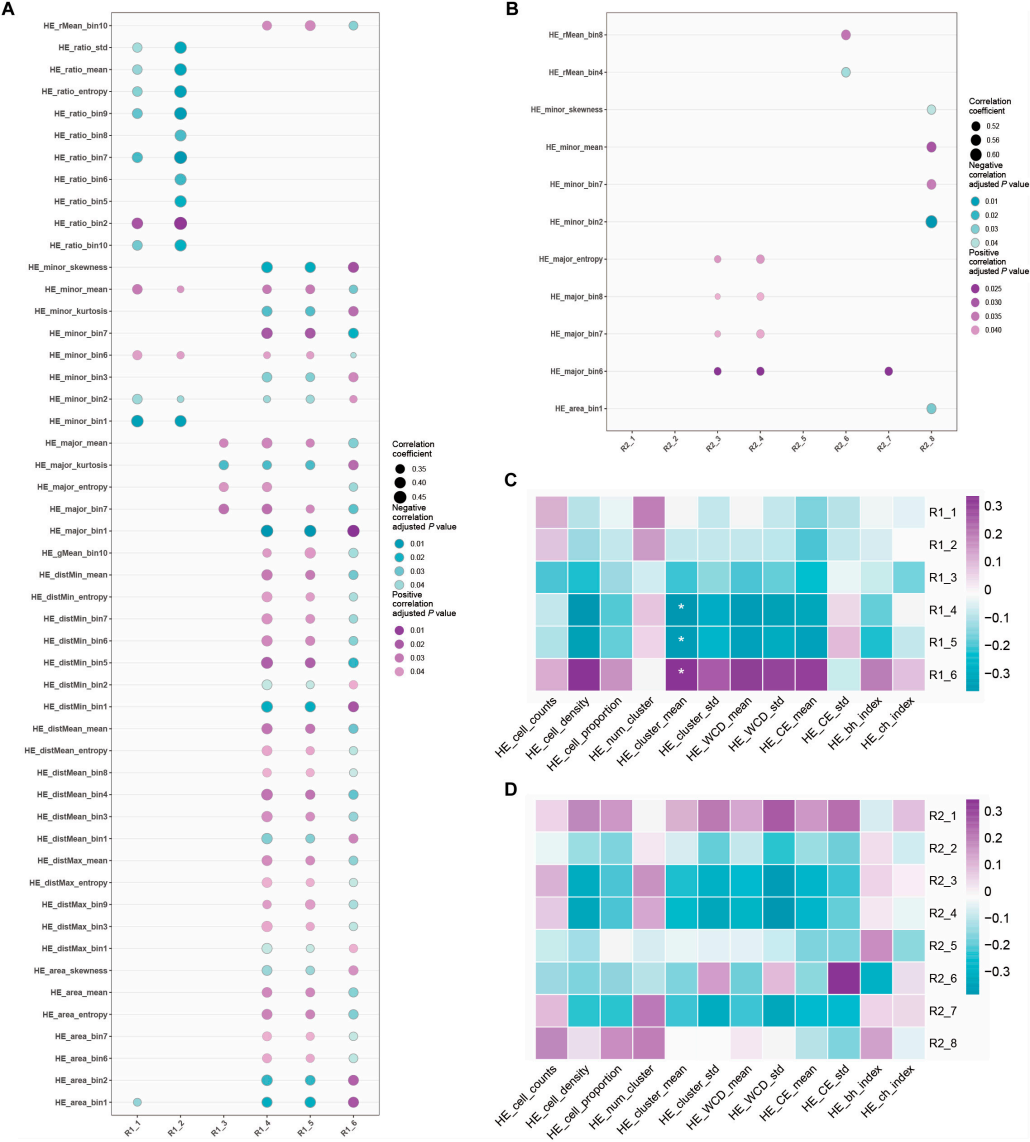
Correlation between cellular features of HE WSI and radiomics features. Bubble plots illustrate the correlation between nuclear features of H&E slides and radiomic features in both the treatment response prediction model (A) and the prognosis prediction model (B). The bubble size represents the strength of the correlation. The correlation heat map shows the relationships between the spatial characteristics of cellular distribution from H&E slides and radiomic features in both the treatment response prediction model (C) and the prognosis prediction model (D). The intensity of the colors reflects the strength of the correlation, with * indicating a *P* value less than 0.05. Purple, positive correlation; green, negative correlation; R1_1, T1C_wavelet-HHL_glcm_Imc2; R1_2, T1C_wavelet-HHL_glcm_MaximumProbability; R1_3, T2_ log-sigma-5-0-mm-3D_glcm_Autocorrelation; R1_4, T2_wavelet-LHL_firstorder_Median; R1_5, T2_wavelet-LHL_glcm_Autocorrelation; R1_6, T2_wavelet-LHL_glcm_ClusterShade; R2_1, T1_wavelet-HLL_glrlm_ShortRunHighGrayLevelEmphasis; R2_2, T1_wavelet-HLL_gldm_LargeDependenceHighGrayLevelEmphasis; R2_3, T1_wavelet-HLH_glrlm_RunEntropy; R2_4, T1_wavelet-HLH_gldm_LargeDependenceLowGrayLevelEmphasis; R2_5, T1C_wavelet-HLL_glcm_Imc1; R2_6, T1C_wavelet-HLH_glcm_DifferenceEntropy; R2_7, T1C_wavelet-HLH_glrlm_RunVariance; R2_8, T2_wavelet-HLH_glcm_ClusterProminence.

To investigate the biological mechanism underlying the radiomic features in the treatment response prediction model, we conducted a Spearman correlation analysis between the 6 selected radiomic features and 150 nuclear morphological features (NMFs) from H&E slides, resulting in 146 significant correlation pairs (Table S8 and Fig. 3A). Among these, T2_wavelet-LHL_firstorder_Median and T2_wavelet-LHL_glcm_ClusterShade exhibited the highest number of correlations with NMF, totaling 78 pairs. Specifically, all correlation pairs showed |*r*| between 0.31 and 0.44 (Fig. 3A). Additionally, we extracted 12 cell spatial distribution features (CSDFs) from H&E slides, which yielded 3 correlation pairs with |*r*| values between 0.34 and 0.36 (Fig. 3C). We also performed Spearman’s rank correlation analyses between radiomic features and IHC-derived NMF (Table S8 and Fig. S2). The results demonstrate moderate correlations between radiomic features and IHC-derived NMF, including CD45RO (69 pairs, |*r*| = 0.31 to 0.48), PD-L1 (73, |*r*| = 0.32 to 0.48), CD8 (84, |*r*| = 0.30 to 0.59), CD163 (53, |*r*| = 0.32 to 0.59), CD68 (23, |*r*| = 0.32 to 0.47), FOXP3 (4, |*r*| = 0.34 to 0.40), Carbonic Anhydrase IX (CAIX) (6, |*r*| = 0.33 to 0.39), CD66b (9, |*r*| = 0.35 to 0.42), and CD19 (19, |*r*| = 0.36 to 0.50). We noted negative correlations in 265 pairs (*r* = −0.30 to -0.59) and positive correlations in 221 pairs (*r* = 0.31 to 0.59). All of these correlations were moderate, with |*r*| values ranging from 0.30 to 0.60.

To uncover the biological basis underlying radiomic features derived from the prognosis prediction model, we conducted a Spearman correlation analysis between 150 H&E-derived NMF and 8 predictive radiomic features (Table S9 and Fig. 3B). Specifically, all of these pairs exhibited |*r*| > 0.41. We also extracted 12 CSDFs from H&E slides; however, there was no correlation with prognostic radiomic features (Fig. 3D). Additionally, we also performed Spearman correlation analysis to assess the association between prognostic radiomic features and IHC-derived NMF (Table S9 and Fig. S2). Significant correlations were identified in all IHC WSIs: PD-L1 (80 pairs, |*r*| = 0.44 to 0.64), CD45RO (65, |*r*| = 0.42 to 0.67), CD19 (35, |*r*| = 0.44 to 0.58), CD66b (61, |*r*| = 0.42 to 0.67), FOXP3 (21, |*r*| = 0.41 to 0.71), CD68 (6, |*r*| = 0.51 to 0.60), CD8 (10, |*r*| = 0.42 to 0.56), CD163 (5, |*r*| = 0.51 to 0.57), and CAIX (2, *r* = −0.52 and 0.53, respectively). Among these correlations, 136 pairs showed negative correlations (*r* = −0.41 to −0.71), while 165 pairs showed positive correlations (*r* = 0.42 to 0.64), with all indicating moderate strength. Among the radiomic features correlated with 12 CSDF from IHC slides (Fig. S3), only one pathological feature (CD68_ch_index) showed a significant correlation with 4 radiomic features (|*r*| = 0.46 to 0.48).

## Discussion

In recent years, the advent of ICIs, including PD-1 inhibitors, has ushered in a new era for cancer immunotherapy. NPC, characterized as a “hot tumor”, is particularly suited for this therapy. Previous studies have shown that biomarkers, such as PD-L1 [18], TMB [20], and tumor-infiltrating lymphocytes (TILs) [30], can reflect the immunological landscape and predict immunotherapy responses in NPC. In particular, PD-L1 expression is a significant predictor in NPC immunotherapy. Studies show that PD-L1-positive patients have higher response rates to ICI, such as PD-1 inhibitors [31]. For instance, a systematic review and meta-analysis found that patients with PD-L1 expression ≥ 1% had higher overall remission rates in R/M NPC, along with better PFS and OS [32]. However, the traditional biomarkers frequently necessitate the use of multiple tumor samples and invasive biopsies, resulting in considerable costs. Thus, the development of accurate and noninvasive biomarkers has become a critical clinical challenge.

Unlike conventional PD-L1 immunohistochemistry, MRI-based radiomics capture microstructural changes in tumor tissue through whole-tumor texture analysis and predict immunotherapy response more accurately than PD-L1 expression alone [29,33]. However, existing models for predicting immunotherapy outcomes and prognoses suffer from poor biological interpretability [25,34,35]. To address these issues, our study introduced the radiomic models specifically designed to predict the response to immunotherapy and the prognosis in patients with LANPC. The noninvasive models showed impressive performance in both training and test cohorts, achieving AUCs of 0.872 and 0.760, respectively. Importantly, it also offered high-accuracy predictions for the prognosis of LANPC patients. Furthermore, the study provided a biological interpretation of the radiomic model with TME quantitative features, enhancing its clinical utility.

Immune cells infiltrating tumors engage in complex and dynamic interactions with tumor cells within the TME, significantly influencing the efficacy of immunotherapeutic approaches and patient prognosis [36]. To clarify the biological interpretability of predictive models, we presented a cross-scale framework linking macroscopic radiological features to pathological features from H&E and IHC staining. This integration of radiomics and microscopic assessments—such as cell density, morphology, and tissue architecture—uncovers the pathobiological basis of model predictions. Cell nuclear feature extraction is a potent tool in TME analysis. It enables precise immune cell localization and quantification while retaining spatial information for interaction studies. Advanced algorithms enhance analytical accuracy, and single-cell analysis capabilities, combined with compatibility with traditional histopathology, allow for a more thorough comprehension of TME complexity.

We extracted morphological features of cell nuclei and then aggregated them into 150 patient-level features and obtained 12 CSDF from H&E WSIs. Our analysis showed that treatment response-related radiomic features exhibited moderate correlations with NMF from H&E WSIs. Notably, the R1_2 (CET1WI_wavelet_HHL_glcm_MaximumProbability) and R1_5 (T2WI_wavelet_LHL_glcm_Autocorrelation) features emerged as the 2 most significant contributors in immunotherapy response modeling. The R1_2 feature reflects the heterogeneity of the tissue structure, the degree of vascularization, and the regularity of tissue arrangement by calculating the highest probability value in the Gray-Level Co-occurrence Matrix (GLCM) within the image. This feature correlates with the ratio of the tumor cells’ nucleus long axis to short axis, which is primarily influenced by nuclear morphology anisotropy, the differentiation status of the tumor cells, and the overall morphological characteristics of the tissue [37], while the R1_5 feature reflects the local consistency of tissue structure, texture correlation, and overall morphological characteristics by calculating the autocorrelation coefficients in the GLCM, thereby assessing the regularity and repetitive patterns of the tissue architecture.

A similar analysis was performed on the 2 features that contributed most significantly to the prognostic models. The R2_4 feature (T1WI_wavelet_HLH_gldm_LargeDependenceLowGrayLevelEmphasis) reflects the intensity of the dependence of texture features of the tumor tissue structure, especially in the region of low gray values. The R2_8 feature (T2WI_wavelet_HLH_glcm_ClusterProminence) quantifies the complexity and heterogeneity of the internal tissue structure of the tumor by quantifying the degree and significance of gray value aggregation within the tumor tissue. Specifically, the characteristics of the gray value aggregation region may be associated with the distribution of tumor cells and the density of the tissue. The R2_4 feature was linked to the major axis of nuclei features, and the R2_8 feature was related to the minor axis of nuclei features of tumor cells. The length of the major axis and minor axis of an ellipse of equal area centered on the cell nucleus can reflect the proliferation status and the degree of abnormality of the cell. Typically, the nuclear morphology of tumor cells changes, often involving enlarged or irregularly shaped nuclei, which may be indicative of the degree of malignancy of the tumor [37]. However, the correlation with CSDF was found to be weak, potentially due to the more microscopic and abstract nature of CSDF compared to NMF and radiomic features.

We also conducted a digital quantification of both NMF and CSDF from WSIs of one hypoxia-related biomarker and 8 immune-related IHC biomarkers. The interaction between PD-L1 and PD-1 on tumor and immune cells inhibits T cell activation, thereby shielding tumors from immune attacks, which represents a key mechanism of immune evasion. Additionally, PD-L1 played a critical role in immune modulation and tolerance [38]. Our study found that some radiomic features associated with treatment response were moderately correlated with PD-L1 NMF. Given the multi-scale nature of our analysis, we recognized that the correlation coefficients may not be high. Nevertheless, these findings suggested that radiomic features might provide noninvasive, complementary insights into the immunological microenvironment, though further validation was warranted [32,39]. Moreover, other immune markers, particularly CD8, CD45RO, and FoxP3, had significant correlations with radiomic features, suggesting their potential to modulate the effects of PD-1 inhibitors. CD8^+^ Tregs could inhibit antitumor immunity through the secretion of cytokines (e.g., interleukin-10 [IL-10] and transforming growth factor-β [TGF-β]) and the engagement of receptors (e.g., PD-1 and CTLA-4), thereby reducing the effectiveness of immunotherapy. They could also have influenced the TME both metabolically and through direct cell contact, resulting in immune suppression and affecting treatment outcomes [40–43]. Conversely, CD45RO^+^ memory T cells, crucial for immune surveillance, effectively targeted NPC tumor cells by detecting and eliminating those displaying aberrant antigens, thus potentially serving as biomarkers for therapeutic response. Our findings aligned with previous research linking variations in CD8^+^ and CD45RO^+^ T cells to patient outcomes.

Furthermore, we investigated the potential relationship between prognostic radiomic features and immunomarkers. A substantial number of statistically significant correlations were observed between prognostic radiomic features and IHC-derived NMF, especially for PD-L1, CD19, CD45RO, CD66b, and FOXP3. The highest correlation coefficient between PD-L1 and radiomic features was 0.64. High PD-L1 expression in NPC was associated with a better prognosis when treated with ICIs (e.g., PD-1/PD-L1 inhibitors) [32]. This was because PD-L1 expression was often a sign of immune evasion in tumors, and blocking this pathway might have enhanced immune response, improving outcomes. Specific subsets of CD19^+^ B cells, such as CD19^+^CD24^+^CD27^+^ and CD19^+^CD24hiCD38hi, secreted IL-10, contributing to immune regulation in the TME [44]. The presence of tertiary lymphoid structures, enriched with CD19^+^ B cells, was associated with NPC progression and response to immunotherapy, indicating their significance in patient prognosis [45]. Accumulation of FoxP3^+^ Tregs in various tumors, including NPC, contributed to immune evasion through the secretion of immunosuppressive molecules such as TGF-β and IL-10, which inhibited the activity of other immune cells and consequently affected patient prognosis [46]. CD66b was likely associated with tumor-associated neutrophils, which played a crucial role in various aspects of tumorigenesis, including tumor development, extracellular matrix remodeling, angiogenesis, cell migration, and immune suppression [45]. The indirect associations of radiomic features with immune infiltrates implied their dual capability in characterizing both tumor heterogeneity and the underlying immune landscape, revealing radiomics’ potential in decoding TME biology. Unlike previous studies [25,26], our study combined both radiomic features and multiple immune markers, enabling a more comprehensive understanding of the tumor immune microenvironment and providing a multidimensional perspective for predicting the efficacy of PD-1 inhibitors and patient prognosis.

Our study has some limitations. First, as a retrospective study, it may have been subject to selection bias. Further prospective studies in endemic and nonendemic regions were necessary to enhance the model’s accuracy and generalizability. However, prospective validation would take several years and could not be achieved at this stage. Second, we collected data from LANPC patients undergoing immunotherapy at 10 hospitals across China. However, since immunotherapy for LANPC was a recent development, this limited our sample size. Furthermore, this study only included patients receiving PD-1 inhibitors with CRT, without a control group. We intended to increase the sample size in subsequent research endeavors and evaluate whether incorporating PD-1 inhibitors offered distinct survival benefits for low- and high-risk groups. Third, we did not include other immunotherapy biomarkers such as tertiary lymphoid structures, TMB, and sequencing data; however, these data were not accessible in clinical settings. Fourth, the clinical translation of radiomic models into clinical use remained challenging. The extraction and analysis of quantitative image features usually required complex computing resources. In the future, efforts should be made to transform the output of radiomic models into forms that are easily comprehensible and acceptable to clinicians, such as risk scoring systems and online web-based prediction tools, to promote the clinical application of these models.

In summary, we developed and validated MRI-based radiomic models that predicted responses to PD-1 inhibitors and outcomes in patients with LANPC. These radiomic models enhanced biological interpretability, shedding light on the underlying biological underpinnings of their predictions and improving their reliability and practical utility. Our MRI-based radiomic models were poised to become valuable tools for refining prognostic stratification and guiding personalized treatment strategies.

## Materials and Methods

### Patients and dataset

Consecutive patients with LANPC who received PD-1 inhibitors combination therapy at 10 academic medical centers between January 2018 and December 2023 were included. The inclusion criteria were as follows: (a) patients with pathologically proven NPC; (b) patients with stage III–IVa according to the 8th edition of the American Joint Committee on Cancer/International Union Against Cancer TNM classification at the initial diagnosis; (c) patients who received PD-1 inhibitors in combination with radiochemotherapy; and (d) patients who underwent MRI scans within 1 month before immunotherapy. Exclusion criteria were as follows: (a) incomplete clinicopathological or clinical follow-up data, (b) with other concurrent tumors, (c) without measurable lesions according to imRECIST v.1.1 criteria [47], (d) lack of baseline MRI data, and (e) poor image quality, incomplete MRI sequences, or lack of follow-up imaging data. Figure S4 shows the inclusion and exclusion flowchart for patients. Given the null hypothesis of an AUC of 0.50 and the alternate hypothesis of an AUC of 0.80, along with a proportion of response rate of 70%–75% [48] and a statistical power of 90%, the minimum required sample size in the external test cohort was determined to be 37 patients. The sample size was computed using PASS 2023, version 23.0.2. Finally, this study included a total of 246 patients, which were assigned to a training cohort (*n* = 117) and an external test cohort (*n* = 129). Feature selection and model development were confined solely to the training cohort, while the validation cohort was reserved exclusively for assessing the model’s generalizability and robustness.

The primary outcome was the response to immunotherapy, assessed using imRECIST V.1.1 criteria [47], which were classified as follows: iCR, iPR, iSD, iCPD, and immune unconfirmed progressive disease (iUPD). Patients with iPR and iCR were categorized as responsive, while those with iSD and iCPD were categorized as non-responsive. The status of iUPD indicated the treatment effect based on subsequent follow-up. The treatment efficacy was evaluated at least 4 weeks after the initiation of treatment. The second outcome was OS, defined as the time from the initiation of immunotherapy to death from any cause.

Clinical information was acquired from the medical records. These data included age, sex, tumor stage, time of initial immunotherapy, WBC count, lymphocyte count, neutrophil count, hemoglobin, and EBV DNA. Tumor stage and laboratory tests were collected before the start of immunotherapy. Data collection and evaluation were completed in January 2025.

### MRI acquisition and image segmentation

**Fig. 4.**
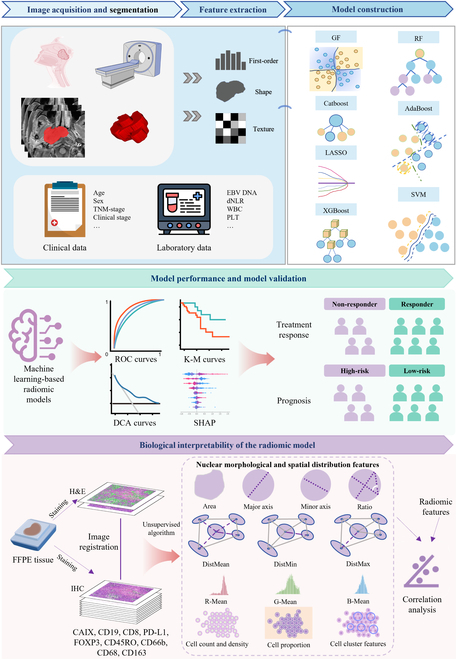
Overall study design. The main steps include MR image acquisition and segmentation, feature extraction, model development, model performance and validation, and biological interpretability of the radiomic model.

The overall design of the study is illustrated in Fig. 4. All MRI images were acquired from the Picture Archiving and Communication System. Detailed MRI acquisition parameters are provided in Tables S10 to S13. The N4 bias correction algorithm and image gray value standardization (0 to 255) were used to minimize the centralization effect from different hospitals and scanners. The images were isotropically resampled to a voxel dimension of 1 × 1 × 1 mm (*x*, *y*, *z*) voxel dimension using the “sitkBSpline” algorithm to normalize the voxel spacing. To reduce noise and discrete intensity, Hounsfield units were set to 25 bins. In the training cohort, 2 radiologists J.S. (Reader 1) and X.W (Reader 2), each with 5 years of experience in head and neck cancer diagnosis, independently delineated the tumors using ITK-SNAP software (version 3.8.0) on T1WI, T2WI, and CET1WI. The segmentation results were carefully reviewed by a senior radiologist, B.Z (Reader 3), with 10 years of experience in head and neck cancer diagnosis. Fifty cases were randomly selected from the training cohort and re-outlined by Reader 1 after a 1-month washout period. The Dice coefficient and Hausdorff distance were applied to evaluate the consistency of tumor segmentation, while the repeatability of extracted features was evaluated using intra-class and inter-class correlation (ICC) coefficients. The segmentation masks for the external test cohort were generated by J.S. to better reflect clinical practice, as suggested by the METRICS tool (Fig. S1) [49].

### Radiomic feature extraction

Radiomic feature extraction was conducted using the open-source Pyradiomics 3.0.1 package. Initially, 107 features were extracted from original images, which included 14 shape-based, 18 histogram-based, 24 GLCM texture, 14 Gray-Level Dependence Matrix texture, 16 Gray-Level Size Zone Matrix texture, 16 Gray-Level Run Length Matrix texture, and 5 Neighborhood Gray Tone Difference Matrix texture features. Additionally, 744 wavelet features were derived through wavelet filtering, and 279 Laplacian of Gaussian (LoG) features were generated from LoG filtering with kernel sizes of 1, 3, and 5. Finally, we extracted 1,130 features per sequence for raw, wavelet-transformed, and LoG-filtered MRI images, resulting in a total of 3,390 (1,130 × 3) extracted features for each patient (Table S14).

### Radiomic feature selection

Considering the segmentation differences between different readers, features with ICC ≥ 0.75 within and between groups were selected as high-reliability features for downstream analysis. The ComBat coordination method was employed to reduce discrepancies in feature distributions across different sources. This method utilizes empirical Bayes frameworks to estimate the parameters of the distributions and correct for systematic biases, ultimately enhancing the reliability of feature selection and radiomic modeling. Subsequently, the mean normalization algorithm was used to further standardize the features [50,51]. In the feature selection process, PCC (|*r*|>0.99) was used to eliminate redundant features, while the ANOVA algorithm was employed to further pinpoint features associated with treatment response to immunotherapy or survival outcomes. Through these multiple stages of feature selection, we ultimately derived the optimal feature subset for subsequent analysis.

Seven machine learning classifiers were compared to construct the radiomic signature (i.e., Radscore), consisting of adaptive boosting, Random Forest, Support Vector Machine, Logistic Regression, Gaussian Process, eXtreme Gradient Boosting, and CatBoost. The algorithm parameters were optimized using 5-fold cross-validation to obtain the optimal radiomic model. Clinical models were built using univariate and multivariate logistic regression analyses based on clinical data. Subsequently, a combined model was constructed by integrating significant clinical variables with the Radscore, utilizing the stepwise multivariate logistic regression.

The predictive performance of the radiomic model was subsequently compared with that of both the clinical and combined models. This comparison was conducted using receiver operating characteristic (ROC) curves, with metrics including the area AUC, accuracy, sensitivity, and specificity. DeLong’s test was used to statistically compare the AUC values between the different models. DCA was conducted to evaluate the clinical net benefit of the models. Additionally, SHAP was employed to visualize and analyze the model’s prediction process [52].

### Development of models for predicting survival outcomes

We selected patients with more than 2 years of follow-up for OS prediction, ultimately including 105 patients in the training cohort and 130 in the external test cohort. To address the imbalance in label distribution within the follow-up dataset, the Synthetic Minority Over-sampling Technique method was used to resample the training data [53].

After resampling, radiomic feature normalization, selection, and modeling were performed following the aforementioned process to develop the optimal model, from which the Radscore associated with OS was derived. A Cox proportional hazards regression model was constructed to calculate the C-index for the radiomic model. Clinical models were constructed using both univariate and multivariate Cox proportional hazards regression analyses based on clinical data. Additionally, a combined model was developed by integrating significant clinical variables with the Radscore using the multivariate Cox regression method.

The potential association between the models and prognosis was evaluated in the training cohort and validated in an external test cohort using Kaplan–Meier survival analysis. The “surv_cutpoint” function from the “survminer” package was utilized to determine the optimal cutoff value according to the risk score of the models, allowing us to categorize patients into high- and low-risk subgroups.

### H&E WSI-derived pathological feature extraction

We collected H&E-stained slides from patients in the external test cohort. The slides were digitized into SVS format at 40× magnification using the Digital Pathology Slide Scanner KF-PRO-400 (Ningbo, Zhejiang, China). Each WSI was manually reviewed by a senior pathologist to identify and exclude artifacts. Only images confirmed to be artifact-free were selected for further analysis. We preprocessed each WSI by applying a pixel threshold to eliminate the white background, defining pixels with a mean RGB channel value exceeding 210 as background. Subsequently, we segmented the non-background areas into non-overlapping 2,048 × 2,048-pixel patches and utilized an unsupervised segmentation algorithm to delineate cell nuclei within these patches. Using the cell segmentation results, we extracted a total of 162 TME quantitative features from each WSI, which can be categorized into 2 main types.

The first category includes 150 NMF. For each segmented nucleus, we first extracted 10 morphological features, comprising the nuclear area, the lengths of the major and minor axes, the aspect ratio (major to minor axis length), and the mean pixel values across the RGB channels. Additionally, we measured the mean, maximum, and minimum distances to neighboring nuclei within the Delaunay triangulation graph. Then for each NMF, we generated a 10-bin histogram and calculated 5 statistical metrics (mean, standard deviation [SD], skewness, kurtosis, and entropy) to aggregate cellular features into a 150-dimensional imaging feature vector for each patient (Table S15). The NMF extraction was performed using MATLAB R2021a.

The second category includes 12 CSDFs (Table S16). First, we calculated 3 basic features: cell counts, cell densities, and cell proportions on a given WSI. Cell densities and proportions were obtained by dividing the cell counts and the total area of cells by the total tissue area, respectively [54]. We then applied the Birch clustering algorithm to identify the cell clusters on the WSI. This algorithm has the advantage of automatically learning the data distribution to yield an optimal number of clusters without the need to pre-define cluster numbers. Based on the clustering results, we calculated a set of cell spatial features, including the number of clusters, mean and SD of cluster size, mean and SD of within-cluster dispersion, mean and SD of cluster extent, the Ball-Hall Index, and the Calinski–Harabasz Index. All CSDF extraction was performed in a Python environment (version 3.8.13).

### IHC WSI-derived TME quantitative feature extraction

All patients from the external test cohort had available formalin-fixed paraffin-embedded (FFPE) tissue samples. FFPE samples were cut into 3-mm-thick sections, which were then processed for IHC staining. Detailed information about IHC staining is provided in the Supplementary Materials. The immune- and hypoxia-related IHC biomarkers included PD-L1, CD8 (cytotoxic T cells), FOXP3 (regulatory T cells), CD19 (B cells), CD45RO (memory T cells), CD66b (macrophages), CD68 and CD163 (tumor-associated macrophage), and CAIX (a marker of hypoxia) (Appendix A1). All the IHC-stained slides were digitized into mrxs format at 40× magnification using the Pannoramic MIDI Scanners (3DHISTECH, Hungary).

We first applied a rigid registration algorithm using the imregister function in the MATLAB environment (version R2021a) to align the IHC-stained slides with their corresponding H&E WSI from the same patient, using the H&E WSI as the fixed image and the IHC slides as the moving images. For each registered IHC-stained slide, we then performed a color deconvolution method to identify the target antigen areas, specifically the gray areas [55]. Subsequently, a total of 150 NMFs and 12 CSDFs were extracted from the registered IHC slides for each IHC marker using the same methods applied to the whole H&E WSIs. Furthermore, we made reference to an associated study in which IHC sections of NPC tissues were used to examine the expression level of PD-L1 and calculate the CPS [56] (Appendix A2).

### Statistical analysis

Statistical analyses were conducted using SPSS 27.0, Python 3.7.6, and R 4.4.0. Statistical comparisons between the 2 groups of continuous variables utilized the *t* test and Wilcoxon rank-sum test, while categorical variables were compared using the chi-square test or Fisher’s exact test. All machine learning algorithms used in the construction of the radiomic model were implemented using the scikit-learn library in Python. We calculated Spearman correlations between radiomic and pathological features, applied false discovery rate correction with a threshold of 0.05 to control for multiple comparisons, and classified correlation coefficients as weak (<0.3), moderate (0.3 to 0.7), or strong (>0.7) [57]. ROC curves were plotted using the “pROC” package. Logistic regression analysis, Kaplan–Meier curve analysis, and calibration curve plotting were performed using the “rms”, “survminer”, and “survival” packages, respectively. Calibration curves were created through bootstrapping with 1,000 resamples and were evaluated using the Hosmer–Lemeshow goodness-of-fit test via the “Resource Selection” package. Furthermore, clinical impact curves and decision curves were plotted using the “rmda” package. The SHAP algorithm was utilized to visually assess the contribution of each feature within the radiomic model. Kaplan–Meier analysis and log-rank test were used for survival analysis and inter-group comparisons, respectively. A 2-sided *P* value <0.05 was considered to be statistically significant.

## Data Availability

Data are available upon reasonable request to the corresponding authors. The raw image, clinical, and follow-up data are not publicly available due to privacy and ethical restrictions.

## Supplementary Materials

Supplementary 1 Tables S1 to S16 Figs. S1 to S6 Appendices A1 and A2 Supplementary Materials
