# Supplementary material for: Radiomic Model Associated with Tumor Microenvironment Predicts Immunotherapy Response and Prognosis in Patients with Locoregionally Advanced Nasopharyngeal Carcinoma
Source: Research (Wash D C). 2025 Jun 24;8:0749. doi: 10.34133/research.0749 (PMC12187091; doi:10.34133/research.0749)

**Supplementary Materials**

**Supplementary Table S1**: Segmentation Difference Analysis

**Supplementary Table S2**: Radiomic Features Associated with Treatment Response

**Supplementary Table S3**: Univariate and Multivariate Logistic Regression Analyses of Clinical Variables Predicting Treatment Response

**Supplementary Table S4**: Selected Radiomic Features for Prognosis

**Supplementary Table S5**: Univariate and Multivariate Cox Regression Analyses of Clinical Variables for Prognosis

**Supplementary Table S6**: Predictive performance of the model for prognosis

**Supplementary Table S7**. Predictive performance of the model for different year prognosis

**Supplementary Figure S1**: METRICS Score of the Study

**Supplementary Table S8**: Correlation Analysis of Radiomic Features Related to Immunotherapy Response with Nuclear Morphology and Hypoxia-Immune-Related Biomarkers from H&E and IHC Slides

**Supplementary Figure S2**: Correlation Between Radiomic Features Related to Immunotherapy Response and

Prognosis with Nuclear Morphological Features from IHC Slides

**Supplementary Table S9**: Correlation Analysis of Radiomic Features Related to Prognosis with Nuclear Morphology and Hypoxia-Immune-Related Biomarkers from H&E and IHC Slides

**Supplementary Figure S3**: Correlation Analysis of Radiomic Features Related to Immunotherapy Response and

Prognosis with 12 Cell Spatial Distribution Features from IHC Slides

**Supplementary Figure S4**: Patient inclusion and exclusion flowchart

**Supplementary Table S10-13**: MRI Image Acquisition Protocols

**Supplementary Table S14**: Extracted Radiomic Features

**Supplementary Table S15**: 150 Patient-Level Nuclear Features Extracted from H&E WSIs.

**Supplementary Table S16**: Description of 10 Types of Nuclear Morphological Features and 12 Cell Spatial

Distribution Features

**Appendix A1**: Detailed Information on IHC Staining

**Appendix A2**: Evaluation of PD-L1 Expression Level

**Supplementary Figure S5**: Clustering of TME Quantitative Features Enriched in Low- and High-risk Groups

**Supplementary Figure S6**: Correlation Between Radiomic Features with Nuclear Morphological Features from

H&E and IHC Slides in the PD-L1 High-Expression Group

**Supplementary Table S1.** Difference analysis of segmentation

|  |  | CET1WI | TIWI | T2WI |
| --- | --- | --- | --- | --- |
| Dice Score | Intra class | 0.975 (0.950-0.985) | 0.975 (0.957-0.985) | 0.973 (0.955-0.986) |
|  | Inter class | 0.840 (0.792-0.886) | 0.809 (0.742-0.839) | 0.823 (0.752-0.856) |
| HD | Intra class | 8.093 (4.840-12.75) | 7.211(5.289-11.93) | 7.416 (4.083-10.99) |
|  | Inter class | 14.75 (9.849-22.59） | 16.99 (11.45-28.36） | 16.35 (10.01-23.41） |
| ICC | Intra class | 0.988 (0.960-0.994） | 0.986 (0.961-0.994） | 0.989 (0.976-0.994） |
|  | Inter class | 0.930 (0.811-0.967） | 0.911 (0.742-0.960） | 0.883 (0.746-0.946） |

**Supplementary Table S2.** The selected radiomic features of treatment response

| R1_1 | CET1WI_wavelet_HHL_glcm_Imc2 |
| --- | --- |
| R1_2 | CET1WI_wavelet_HHL_glcm_MaximumProbability |
| R1_3 | T2WI_log_sigma_5_0_mm_3D_glcm_Autocorrelation |
| R1_4 | T2WI_wavelet_LHL_firstorder_Median |
| R1_5 | T2WI_wavelet_LHL_glcm_Autocorrelation |
| R1_6 | T2WI_wavelet_LHL_glcm_ClusterShade |

**Supplementary Ta**b**le S3.** Univariate and multivariate logistic regression analysis for the clinical variables of treatment response

|  | Univariate analysis | | | Multivariate analysis | | |
| --- | --- | --- | --- | --- | --- | --- |
| Variables | OR | 95%CI | P -value | OR | 95%CI | P -value |
| Sex | 2.77 | 0.959 -10.084 | 0.082 |  |  |  |
| Age | 0.975 | 0.939 - 1.01 | 0.161 |  |  |  |
| T stage | 0.691 | 0.396 - 1.144 | 0.169 |  |  |  |
| **N stage** | **1.772** | **1.115 - 2.889** | **0.017*** | **1.594** | **0.977 - 2.598** | **0.062** |
| Clinical stages | 0.799 | 0.386 - 1.56 | 0.525 |  |  |  |
| EB/DNA | 1.537 | 0.669 - 3.599 | 0.314 |  |  |  |
| Neutrophils (10^9^/L)  (10^9^/L) | 1.348 | 1.024 - 1.85 | 0.046* |  |  |  |
| Lymphocytes(10^9^/L)c  (10^9^/L) | 0.921 | 0.683 - 1.219 | 0.501 |  |  |  |
| NLR | 1.034 | 0.925 - 1.222 | 0.619 |  |  |  |
| **WBC (10^9^/L)**  **(10^9^/L)** | **1.368** | **1.073 - 1.798** | **0.017*** | **1.305** | **1.000 - 1.704** | **0.050** |
| Hemoglobin (g/L)  (g/L) | 1.016 | 0.992 - 1.041 | 0.198 |  |  |  |
| Platelets (10^9^/L)  (10^9^/L) | 1.002 | 0.998 - 1.008 | 0.324 |  |  |  |
| Immunotherapy Regimen | 0.867 | 0.566-1.340 | 0.514 |  |  |  |

Note: dNLR, derived neutrophil-to-lymphocyte ratio; WBC, white blood cells.

**Supplementary Table S4.** The selected radiomic features of prognosis

| R2_1 | T1WI_wavelet_HLL_glrlm_ShortRunHighGrayLevelEmphasis |
| --- | --- |
| R2_2 | T1WI_wavelet_HLL_gldm_LargeDependenceHighGrayLevelEmphasis |
| R2_3 | T1WI_wavelet_HLH_glrlm_RunEntropy |
| R2_4 | T1WI_wavelet_HLH_gldm_LargeDependenceLowGrayLevelEmphasis |
| R2_5 | CET1WI_wavelet_HLL_glcm_Imc1 |
| R2_6 | CET1WI_wavelet_HLH_glcm_DifferenceEntropy |
| R2_7 | CET1WI_wavelet_HLH_glrlm_RunVariance |
| R2_8 | T2WI_wavelet_HLH_glcm_ClusterProminence |

**Supplementary Table S5.** Univariate and multivariate Cox analyses for the clinical variables of prognosis

|  | Univariate analysis | | | Multivariate analysis | | |
| --- | --- | --- | --- | --- | --- | --- |
| Variables | HR | 95%CI | P -value | HR | 95%CI | P -value |
| Sex | 1.268 | 0.551-2.922 | 0.577 |  |  |  |
| **Age** | **1.052** | **1.018-1.087** | **0.003*** | **1.046** | **1.012-1.081** | **0.008*** |
| T stage | 1.218 | 0.763-1.946 | 0.409 |  |  |  |
| **N stage** | **1.606** | **1.057-2.440** | **0.026*** | **1.620** | **1.024-2.561** | **0.039*** |
| Clinical stages | 1.010 | 0.546-1.869 | 0.974 |  |  |  |
| EB/DNA | 1.190 | 0.550-2.576 | 0.658 |  |  |  |
| Neutrophils (10^9^/L)  (10^9^/L) | 0.953 | 0.762-1.193 | 0.674 |  |  |  |
| Lymphocytes(10^9^/L) | 1.239 | 0.982-1.563 | 0.071 |  |  |  |
| NLR | 0.990 | 0.905-1.082 | 0.818 |  |  |  |
| WBC (10^9^/L)  (10^9^/L) | 0.830 | 0.662-1.040 | 0.106 |  |  |  |
| **Hemoglobin (g/L)**  **(g/L)** | **0.975** | **0.958-0.993** | **0.006*** | **0.981** | **0.964-0.999** | **0.044*** |
| Platelets (10^9^/L)  (10^9^/L) | 0.999 | 0.995-1.004 | 0.826 |  |  |  |
| **Immunotherapy Regimen** | 1.397 | 0.944-2.069 | 0.095 |  |  |  |

Note: dNLR, derived neutrophil-to-lymphocyte ratio; WBC, white blood cells.

**Supplementary Table S6.** Predictive performance of the model for prognosis

|  |  | C index | 95%CI |
| --- | --- | --- | --- |
| **Training cohort** | Clinical model | 0.723 | 0.688-0.758 |
|  | Radiomic model | 0.860 | 0.844-0.876 |
|  | **Combine model** | **0.850** | **0.821-0.879** |
| **External test cohort** | Clinical model | 0.664 | 0.621-0.707 |
|  | Radiomic model | 0.812 | 0.783-0.841 |
|  | **Combine model** | **0.858** | **0.842-0.874** |

**Supplementary Table S7.** Predictive performance of the model for different year prognosis

|  | Datasets | 1 year-C index | 2 year-C index | 3 year-C index |
| --- | --- | --- | --- | --- |
| Clinical model | Training cohort | 0.625 (0.398-0.831) | 0.721(0.583-0.844) | 0.721(0.599-0.824) |
|  | External test cohort | 0.636 (0.465-0.802) | 0.655(0.495-0.805) | 0.667(0.520-0.808) |
| Radiomic model | Training cohort | 0.864 (0.772-0.942) | 0.908 (0.849-0.956) | 0.899 (0.843-0.958) |
|  | External test cohort | 0.806 (0.694-0.910) | 0.815 (0.709-0.910) | 0.814 (0.717-0.904) |
| Combine model | Training cohort | 0.836 (0.736-0.923) | 0.896 (0.836-0.951) | 0.883(0.822-0.940) |
|  | External test cohort | 0.841(0.735-0.931) | 0.854 (0.752-0.935) | 0.861(0.769-0.938) |

**Supplementary Figure S1.** METRICS score of the study


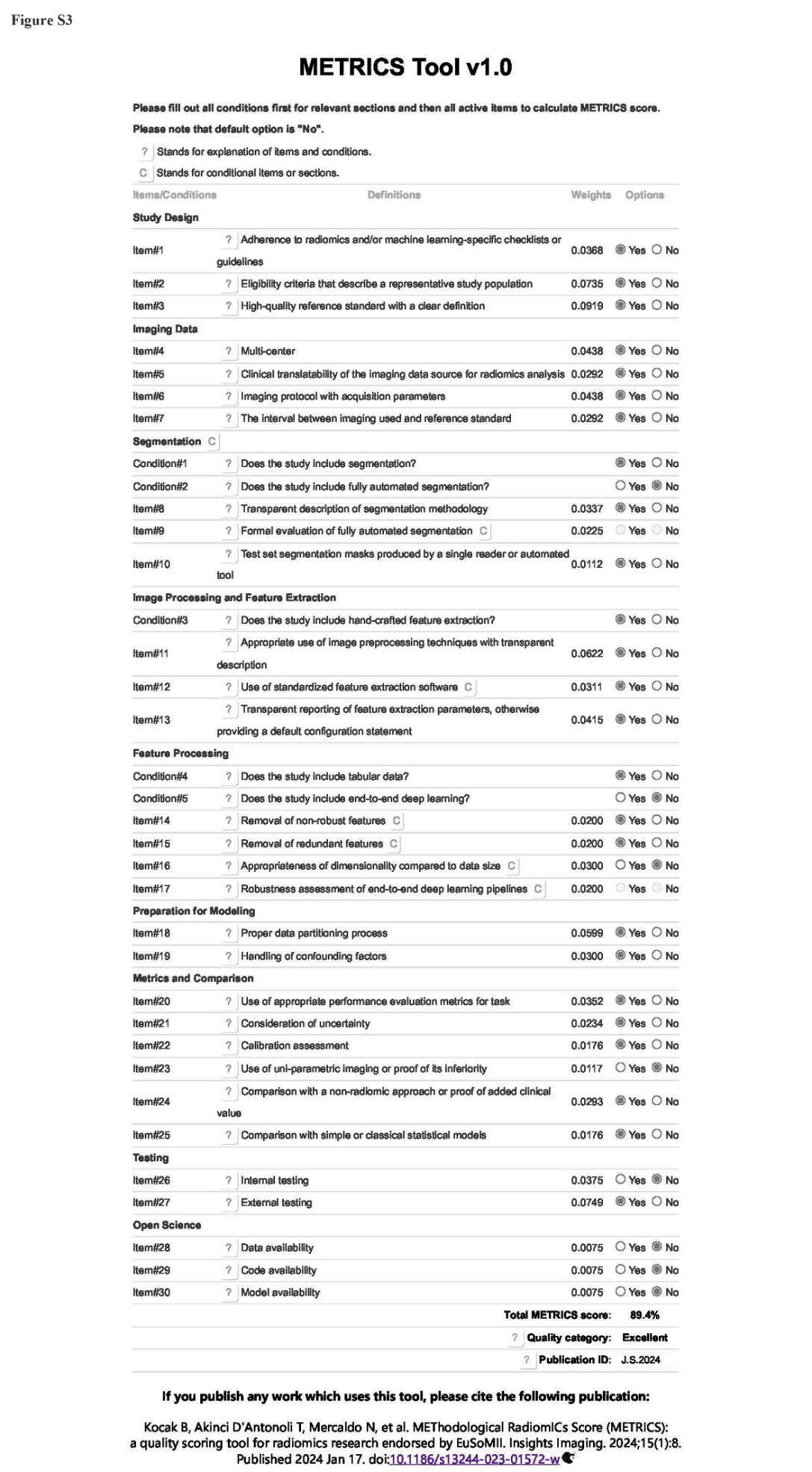


**
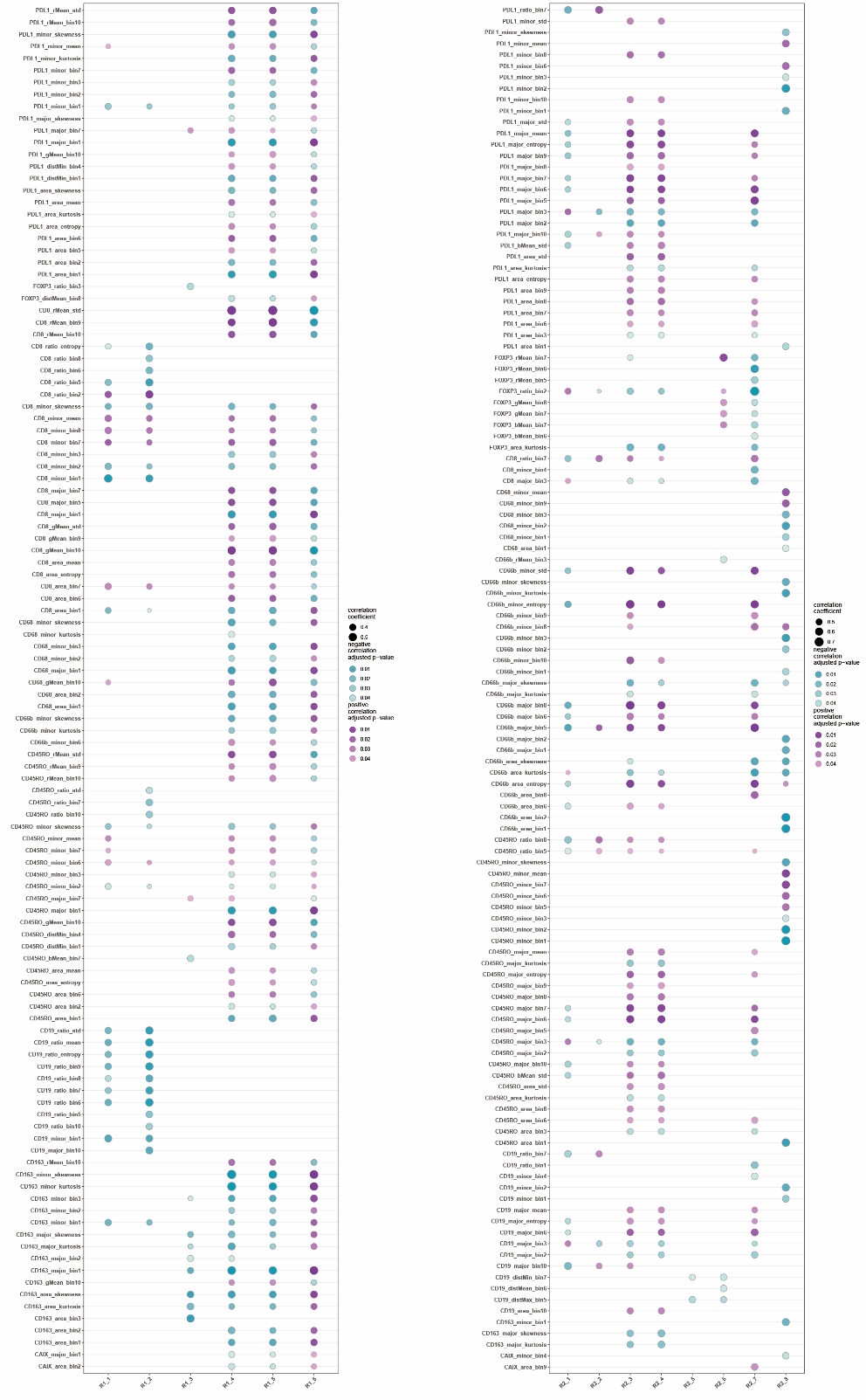
Supplementary Figure S2.** Correlation between radiomic features related to immunotherapy response and prognosis with nuclear morphological features from IHC slides

**Supplementary Table S8.** Correlation analysis of radiomics features related to ICIs response with nuclear morphology and hypoxia-immune-related biomarkers from HE&IHC slides

| Radiomics features | Pathological features | ρ | P value | Calibrated P value |
| --- | --- | --- | --- | --- |
| R1_1 | PDL1_minor_bin1 | -0.378 | 0.010 | 0.029 |
| R1_1 | PDL1_minor_mean | 0.315 | 0.033 | 0.050 |
| R1_1 | CD8_area_bin1 | -0.360 | 0.014 | 0.021 |
| R1_1 | CD8_area_bin7 | 0.399 | 0.006 | 0.034 |
| R1_1 | CD8_minor_bin1 | -0.507 | 0.000 | 0.002 |
| R1_1 | CD8_minor_bin2 | -0.405 | 0.005 | 0.022 |
| R1_1 | CD8_minor_bin7 | 0.377 | 0.010 | 0.016 |
| R1_1 | CD8_minor_bin8 | 0.406 | 0.005 | 0.028 |
| R1_1 | CD8_ratio_bin2 | 0.407 | 0.005 | 0.015 |
| R1_1 | CD8_ratio_bin5 | -0.388 | 0.008 | 0.023 |
| R1_1 | CD8_minor_mean | 0.409 | 0.005 | 0.025 |
| R1_1 | CD8_minor_skewness | -0.375 | 0.010 | 0.018 |
| R1_1 | CD8_ratio_entropy | -0.354 | 0.016 | 0.048 |
| R1_1 | CD19_minor_bin1 | -0.451 | 0.002 | 0.010 |
| R1_1 | CD19_ratio_bin6 | -0.406 | 0.005 | 0.015 |
| R1_1 | CD19_ratio_bin7 | -0.380 | 0.009 | 0.028 |
| R1_1 | CD19_ratio_bin8 | -0.363 | 0.013 | 0.039 |
| R1_1 | CD19_ratio_bin9 | -0.401 | 0.006 | 0.017 |
| R1_1 | CD19_ratio_mean | -0.407 | 0.005 | 0.015 |
| R1_1 | CD19_ratio_std | -0.397 | 0.006 | 0.019 |
| R1_1 | CD19_ratio_entropy | -0.393 | 0.007 | 0.021 |
| R1_1 | CD68_gMean_bin10 | 0.322 | 0.029 | 0.044 |
| R1_1 | CD163_minor_bin1 | -0.396 | 0.006 | 0.018 |
| R1_1 | CD45RO_minor_bin2 | -0.366 | 0.012 | 0.046 |
| R1_1 | CD45RO_minor_bin6 | 0.365 | 0.013 | 0.043 |
| R1_1 | CD45RO_minor_bin7 | 0.317 | 0.032 | 0.048 |
| R1_1 | CD45RO_minor_mean | 0.353 | 0.016 | 0.034 |
| R1_1 | CD45RO_minor_skewness | -0.362 | 0.014 | 0.027 |
| R1_1 | HE_area_bin1 | -0.329 | 0.025 | 0.038 |
| R1_1 | HE_minor_bin1 | -0.437 | 0.002 | 0.011 |
| R1_1 | HE_minor_bin2 | -0.368 | 0.012 | 0.040 |
| R1_1 | HE_minor_bin6 | 0.353 | 0.016 | 0.044 |
| R1_1 | HE_ratio_bin2 | 0.399 | 0.006 | 0.018 |
| R1_1 | HE_ratio_bin7 | -0.385 | 0.008 | 0.025 |
| R1_1 | HE_ratio_bin9 | -0.377 | 0.010 | 0.029 |
| R1_1 | HE_ratio_bin10 | -0.373 | 0.011 | 0.032 |
| R1_1 | HE_minor_mean | 0.367 | 0.012 | 0.031 |
| R1_1 | HE_ratio_mean | -0.364 | 0.013 | 0.039 |
| R1_1 | HE_ratio_std | -0.360 | 0.014 | 0.042 |
| R1_1 | HE_ratio_entropy | -0.368 | 0.012 | 0.035 |
| R1_2 | PDL1_minor_bin1 | -0.337 | 0.022 | 0.029 |
| R1_2 | CD8_area_bin1 | -0.302 | 0.041 | 0.050 |
| R1_2 | CD8_area_bin7 | 0.350 | 0.017 | 0.034 |
| R1_2 | CD8_minor_bin1 | -0.454 | 0.002 | 0.005 |
| R1_2 | CD8_minor_bin2 | -0.343 | 0.020 | 0.024 |
| R1_2 | CD8_minor_bin7 | 0.342 | 0.020 | 0.024 |
| R1_2 | CD8_minor_bin8 | 0.360 | 0.014 | 0.028 |
| R1_2 | CD8_ratio_bin2 | 0.473 | 0.001 | 0.005 |
| R1_2 | CD8_ratio_bin5 | -0.469 | 0.001 | 0.006 |
| R1_2 | CD8_ratio_bin6 | -0.424 | 0.003 | 0.020 |
| R1_2 | CD8_ratio_bin8 | -0.412 | 0.004 | 0.027 |
| R1_2 | CD8_minor_mean | 0.362 | 0.013 | 0.025 |
| R1_2 | CD8_minor_skewness | -0.395 | 0.007 | 0.018 |
| R1_2 | CD8_ratio_entropy | -0.430 | 0.003 | 0.017 |
| R1_2 | CD19_major_bin10 | -0.444 | 0.002 | 0.012 |
| R1_2 | CD19_minor_bin1 | -0.423 | 0.003 | 0.010 |
| R1_2 | CD19_ratio_bin5 | -0.415 | 0.004 | 0.025 |
| R1_2 | CD19_ratio_bin6 | -0.494 | 0.000 | 0.003 |
| R1_2 | CD19_ratio_bin7 | -0.465 | 0.001 | 0.007 |
| R1_2 | CD19_ratio_bin8 | -0.444 | 0.002 | 0.012 |
| R1_2 | CD19_ratio_bin9 | -0.485 | 0.001 | 0.004 |
| R1_2 | CD19_ratio_bin10 | -0.404 | 0.005 | 0.032 |
| R1_2 | CD19_ratio_mean | -0.496 | 0.000 | 0.003 |
| R1_2 | CD19_ratio_std | -0.480 | 0.001 | 0.004 |
| R1_2 | CD19_ratio_entropy | -0.479 | 0.001 | 0.005 |
| R1_2 | CD163_minor_bin1 | -0.350 | 0.017 | 0.020 |
| R1_2 | CD45RO_minor_bin2 | -0.306 | 0.038 | 0.046 |
| R1_2 | CD45RO_minor_bin6 | 0.310 | 0.036 | 0.043 |
| R1_2 | CD45RO_ratio_bin7 | -0.415 | 0.004 | 0.025 |
| R1_2 | CD45RO_ratio_bin10 | -0.407 | 0.005 | 0.030 |
| R1_2 | CD45RO_ratio_std | -0.394 | 0.007 | 0.041 |
| R1_2 | CD45RO_minor_skewness | -0.316 | 0.033 | 0.039 |
| R1_2 | HE_minor_bin1 | -0.419 | 0.004 | 0.011 |
| R1_2 | HE_minor_bin2 | -0.315 | 0.033 | 0.040 |
| R1_2 | HE_minor_bin6 | 0.320 | 0.030 | 0.044 |
| R1_2 | HE_ratio_bin2 | 0.459 | 0.001 | 0.008 |
| R1_2 | HE_ratio_bin5 | -0.427 | 0.003 | 0.018 |
| R1_2 | HE_ratio_bin6 | -0.417 | 0.004 | 0.024 |
| R1_2 | HE_ratio_bin7 | -0.463 | 0.001 | 0.007 |
| R1_2 | HE_ratio_bin8 | -0.414 | 0.004 | 0.025 |
| R1_2 | HE_ratio_bin9 | -0.453 | 0.002 | 0.009 |
| R1_2 | HE_ratio_bin10 | -0.427 | 0.003 | 0.018 |
| R1_2 | HE_minor_mean | 0.314 | 0.034 | 0.040 |
| R1_2 | HE_ratio_mean | -0.440 | 0.002 | 0.013 |
| R1_2 | HE_ratio_std | -0.437 | 0.002 | 0.014 |
| R1_2 | HE_ratio_entropy | -0.446 | 0.002 | 0.011 |
| R1_3 | PDL1_major_bin7 | 0.343 | 0.019 | 0.039 |
| R1_3 | FOXP3_ratio_bin3 | -0.396 | 0.006 | 0.039 |
| R1_3 | CD163_area_bin3 | -0.480 | 0.001 | 0.004 |
| R1_3 | CD163_major_bin1 | -0.380 | 0.009 | 0.014 |
| R1_3 | CD163_major_bin2 | -0.381 | 0.009 | 0.048 |
| R1_3 | CD163_minor_bin3 | -0.317 | 0.032 | 0.048 |
| R1_3 | CD163_area_skewness | -0.414 | 0.004 | 0.006 |
| R1_3 | CD163_major_skewness | -0.366 | 0.012 | 0.020 |
| R1_3 | CD163_area_kurtosis | -0.423 | 0.003 | 0.020 |
| R1_3 | CD163_major_kurtosis | -0.330 | 0.025 | 0.038 |
| R1_3 | CD45RO_major_bin7 | 0.344 | 0.019 | 0.050 |
| R1_3 | CD45RO_bMean_bin7 | -0.396 | 0.006 | 0.039 |
| R1_3 | HE_major_bin7 | 0.371 | 0.011 | 0.028 |
| R1_3 | HE_major_mean | 0.341 | 0.020 | 0.035 |
| R1_3 | HE_major_kurtosis | -0.362 | 0.013 | 0.026 |
| R1_3 | HE_major_entropy | 0.353 | 0.016 | 0.040 |
| R1_4 | PDL1_area_bin1 | -0.450 | 0.002 | 0.003 |
| R1_4 | PDL1_area_bin2 | -0.397 | 0.006 | 0.020 |
| R1_4 | PDL1_area_bin5 | 0.356 | 0.015 | 0.042 |
| R1_4 | PDL1_area_bin6 | 0.385 | 0.008 | 0.016 |
| R1_4 | PDL1_major_bin1 | -0.476 | 0.001 | 0.003 |
| R1_4 | PDL1_major_bin7 | 0.362 | 0.013 | 0.039 |
| R1_4 | PDL1_minor_bin1 | -0.336 | 0.023 | 0.029 |
| R1_4 | PDL1_minor_bin2 | -0.373 | 0.011 | 0.021 |
| R1_4 | PDL1_minor_bin3 | -0.371 | 0.011 | 0.034 |
| R1_4 | PDL1_minor_bin7 | 0.398 | 0.006 | 0.018 |
| R1_4 | PDL1_rMean_bin10 | 0.380 | 0.009 | 0.021 |
| R1_4 | PDL1_gMean_bin10 | 0.340 | 0.021 | 0.041 |
| R1_4 | PDL1_distMin_bin1 | -0.415 | 0.004 | 0.015 |
| R1_4 | PDL1_distMin_bin4 | 0.372 | 0.011 | 0.039 |
| R1_4 | PDL1_area_mean | 0.386 | 0.008 | 0.025 |
| R1_4 | PDL1_minor_mean | 0.356 | 0.015 | 0.037 |
| R1_4 | PDL1_rMean_std | 0.413 | 0.004 | 0.012 |
| R1_4 | PDL1_area_skewness | -0.399 | 0.006 | 0.021 |
| R1_4 | PDL1_major_skewness | -0.342 | 0.020 | 0.049 |
| R1_4 | PDL1_minor_skewness | -0.458 | 0.001 | 0.006 |
| R1_4 | PDL1_area_kurtosis | -0.361 | 0.014 | 0.049 |
| R1_4 | PDL1_minor_kurtosis | -0.416 | 0.004 | 0.015 |
| R1_4 | PDL1_area_entropy | 0.379 | 0.009 | 0.034 |
| R1_4 | CAIX_area_bin2 | -0.391 | 0.007 | 0.044 |
| R1_4 | CAIX_major_bin1 | -0.372 | 0.011 | 0.048 |
| R1_4 | CD8_area_bin1 | -0.408 | 0.005 | 0.013 |
| R1_4 | CD8_area_bin6 | 0.398 | 0.006 | 0.016 |
| R1_4 | CD8_area_bin7 | 0.342 | 0.020 | 0.034 |
| R1_4 | CD8_major_bin1 | -0.471 | 0.001 | 0.004 |
| R1_4 | CD8_major_bin5 | 0.412 | 0.004 | 0.010 |
| R1_4 | CD8_major_bin7 | 0.402 | 0.006 | 0.011 |
| R1_4 | CD8_minor_bin2 | -0.378 | 0.009 | 0.022 |
| R1_4 | CD8_minor_bin3 | -0.389 | 0.007 | 0.028 |
| R1_4 | CD8_minor_bin7 | 0.382 | 0.009 | 0.016 |
| R1_4 | CD8_minor_bin8 | 0.335 | 0.023 | 0.028 |
| R1_4 | CD8_rMean_bin9 | 0.454 | 0.002 | 0.003 |
| R1_4 | CD8_rMean_bin10 | 0.406 | 0.005 | 0.012 |
| R1_4 | CD8_gMean_bin9 | 0.340 | 0.021 | 0.042 |
| R1_4 | CD8_gMean_bin10 | 0.507 | 0.000 | 0.001 |
| R1_4 | CD8_area_mean | 0.374 | 0.010 | 0.030 |
| R1_4 | CD8_minor_mean | 0.351 | 0.017 | 0.025 |
| R1_4 | CD8_rMean_std | 0.563 | 0.000 | 0.000 |
| R1_4 | CD8_gMean_std | 0.383 | 0.009 | 0.017 |
| R1_4 | CD8_minor_skewness | -0.398 | 0.006 | 0.018 |
| R1_4 | CD8_area_entropy | 0.388 | 0.008 | 0.024 |
| R1_4 | FOXP3_distMean_bin8 | -0.381 | 0.009 | 0.042 |
| R1_4 | CD68_area_bin1 | -0.458 | 0.001 | 0.008 |
| R1_4 | CD68_area_bin2 | -0.428 | 0.003 | 0.015 |
| R1_4 | CD68_major_bin1 | -0.470 | 0.001 | 0.006 |
| R1_4 | CD68_minor_bin2 | -0.364 | 0.013 | 0.037 |
| R1_4 | CD68_minor_bin3 | -0.432 | 0.003 | 0.009 |
| R1_4 | CD68_gMean_bin10 | 0.365 | 0.013 | 0.025 |
| R1_4 | CD68_minor_skewness | -0.453 | 0.002 | 0.010 |
| R1_4 | CD68_minor_kurtosis | -0.385 | 0.008 | 0.049 |
| R1_4 | CD163_area_bin1 | -0.403 | 0.006 | 0.011 |
| R1_4 | CD163_area_bin2 | -0.425 | 0.003 | 0.018 |
| R1_4 | CD163_major_bin1 | -0.532 | 0.000 | 0.001 |
| R1_4 | CD163_major_bin2 | -0.353 | 0.016 | 0.048 |
| R1_4 | CD163_minor_bin1 | -0.353 | 0.016 | 0.020 |
| R1_4 | CD163_minor_bin2 | -0.369 | 0.012 | 0.029 |
| R1_4 | CD163_minor_bin3 | -0.405 | 0.005 | 0.010 |
| R1_4 | CD163_rMean_bin10 | 0.385 | 0.008 | 0.023 |
| R1_4 | CD163_gMean_bin10 | 0.348 | 0.018 | 0.036 |
| R1_4 | CD163_area_skewness | -0.448 | 0.002 | 0.006 |
| R1_4 | CD163_major_skewness | -0.418 | 0.004 | 0.020 |
| R1_4 | CD163_minor_skewness | -0.587 | 0.000 | 0.000 |
| R1_4 | CD163_area_kurtosis | -0.362 | 0.014 | 0.020 |
| R1_4 | CD163_major_kurtosis | -0.449 | 0.002 | 0.011 |
| R1_4 | CD163_minor_kurtosis | -0.565 | 0.000 | 0.000 |
| R1_4 | CD66b_minor_bin6 | 0.389 | 0.008 | 0.037 |
| R1_4 | CD66b_minor_skewness | -0.406 | 0.005 | 0.014 |
| R1_4 | CD66b_minor_kurtosis | -0.367 | 0.012 | 0.027 |
| R1_4 | CD45RO_area_bin1 | -0.397 | 0.006 | 0.013 |
| R1_4 | CD45RO_area_bin2 | -0.377 | 0.010 | 0.045 |
| R1_4 | CD45RO_area_bin6 | 0.366 | 0.012 | 0.028 |
| R1_4 | CD45RO_major_bin1 | -0.478 | 0.001 | 0.002 |
| R1_4 | CD45RO_major_bin7 | 0.348 | 0.018 | 0.050 |
| R1_4 | CD45RO_minor_bin2 | -0.311 | 0.036 | 0.046 |
| R1_4 | CD45RO_minor_bin3 | -0.355 | 0.016 | 0.046 |
| R1_4 | CD45RO_minor_bin6 | 0.326 | 0.027 | 0.043 |
| R1_4 | CD45RO_minor_bin7 | 0.378 | 0.010 | 0.035 |
| R1_4 | CD45RO_rMean_bin9 | 0.356 | 0.015 | 0.033 |
| R1_4 | CD45RO_rMean_bin10 | 0.356 | 0.015 | 0.035 |
| R1_4 | CD45RO_gMean_bin10 | 0.416 | 0.004 | 0.009 |
| R1_4 | CD45RO_distMin_bin1 | -0.396 | 0.006 | 0.034 |
| R1_4 | CD45RO_distMin_bin4 | 0.413 | 0.004 | 0.021 |
| R1_4 | CD45RO_area_mean | 0.367 | 0.012 | 0.038 |
| R1_4 | CD45RO_minor_mean | 0.346 | 0.019 | 0.034 |
| R1_4 | CD45RO_rMean_std | 0.424 | 0.003 | 0.010 |
| R1_4 | CD45RO_minor_skewness | -0.387 | 0.008 | 0.027 |
| R1_4 | CD45RO_area_entropy | 0.373 | 0.011 | 0.041 |
| R1_4 | HE_area_bin1 | -0.382 | 0.009 | 0.018 |
| R1_4 | HE_area_bin2 | -0.398 | 0.006 | 0.022 |
| R1_4 | HE_area_bin6 | 0.350 | 0.017 | 0.047 |
| R1_4 | HE_area_bin7 | 0.347 | 0.018 | 0.049 |
| R1_4 | HE_major_bin1 | -0.439 | 0.002 | 0.006 |
| R1_4 | HE_major_bin7 | 0.373 | 0.011 | 0.028 |
| R1_4 | HE_minor_bin2 | -0.319 | 0.031 | 0.040 |
| R1_4 | HE_minor_bin3 | -0.369 | 0.012 | 0.035 |
| R1_4 | HE_minor_bin6 | 0.315 | 0.033 | 0.044 |
| R1_4 | HE_minor_bin7 | 0.396 | 0.006 | 0.019 |
| R1_4 | HE_rMean_bin10 | 0.350 | 0.017 | 0.036 |
| R1_4 | HE_gMean_bin10 | 0.339 | 0.021 | 0.043 |
| R1_4 | HE_distMean_bin1 | -0.392 | 0.007 | 0.035 |
| R1_4 | HE_distMean_bin3 | 0.387 | 0.008 | 0.038 |
| R1_4 | HE_distMean_bin4 | 0.404 | 0.005 | 0.029 |
| R1_4 | HE_distMean_bin8 | 0.340 | 0.021 | 0.050 |
| R1_4 | HE_distMax_bin1 | -0.381 | 0.009 | 0.049 |
| R1_4 | HE_distMax_bin3 | 0.385 | 0.008 | 0.047 |
| R1_4 | HE_distMax_bin9 | 0.338 | 0.022 | 0.043 |
| R1_4 | HE_distMin_bin1 | -0.413 | 0.004 | 0.018 |
| R1_4 | HE_distMin_bin2 | -0.375 | 0.010 | 0.049 |
| R1_4 | HE_distMin_bin5 | 0.415 | 0.004 | 0.022 |
| R1_4 | HE_distMin_bin6 | 0.388 | 0.008 | 0.036 |
| R1_4 | HE_distMin_bin7 | 0.383 | 0.009 | 0.040 |
| R1_4 | HE_area_mean | 0.368 | 0.012 | 0.036 |
| R1_4 | HE_major_mean | 0.378 | 0.010 | 0.035 |
| R1_4 | HE_minor_mean | 0.344 | 0.019 | 0.031 |
| R1_4 | HE_distMean_mean | 0.386 | 0.008 | 0.030 |
| R1_4 | HE_distMax_mean | 0.375 | 0.010 | 0.037 |
| R1_4 | HE_distMin_mean | 0.389 | 0.008 | 0.031 |
| R1_4 | HE_area_skewness | -0.374 | 0.010 | 0.039 |
| R1_4 | HE_minor_skewness | -0.407 | 0.005 | 0.017 |
| R1_4 | HE_major_kurtosis | -0.350 | 0.017 | 0.026 |
| R1_4 | HE_minor_kurtosis | -0.380 | 0.009 | 0.026 |
| R1_4 | HE_area_entropy | 0.377 | 0.010 | 0.035 |
| R1_4 | HE_major_entropy | 0.357 | 0.015 | 0.040 |
| R1_4 | HE_distMean_entropy | 0.370 | 0.011 | 0.047 |
| R1_4 | HE_distMax_entropy | 0.368 | 0.012 | 0.049 |
| R1_4 | HE_distMin_entropy | 0.374 | 0.010 | 0.043 |
| R1_5 | PDL1_area_bin1 | -0.469 | 0.001 | 0.003 |
| R1_5 | PDL1_area_bin2 | -0.377 | 0.010 | 0.020 |
| R1_5 | PDL1_area_bin5 | 0.341 | 0.021 | 0.042 |
| R1_5 | PDL1_area_bin6 | 0.385 | 0.008 | 0.016 |
| R1_5 | PDL1_major_bin1 | -0.461 | 0.001 | 0.003 |
| R1_5 | PDL1_major_bin7 | 0.317 | 0.032 | 0.048 |
| R1_5 | PDL1_minor_bin1 | -0.358 | 0.014 | 0.029 |
| R1_5 | PDL1_minor_bin2 | -0.390 | 0.007 | 0.021 |
| R1_5 | PDL1_minor_bin3 | -0.350 | 0.017 | 0.034 |
| R1_5 | PDL1_minor_bin7 | 0.381 | 0.009 | 0.018 |
| R1_5 | PDL1_rMean_bin10 | 0.415 | 0.004 | 0.021 |
| R1_5 | PDL1_gMean_bin10 | 0.374 | 0.010 | 0.041 |
| R1_5 | PDL1_distMin_bin1 | -0.396 | 0.006 | 0.015 |
| R1_5 | PDL1_distMin_bin4 | 0.343 | 0.019 | 0.039 |
| R1_5 | PDL1_area_mean | 0.366 | 0.012 | 0.025 |
| R1_5 | PDL1_minor_mean | 0.357 | 0.015 | 0.037 |
| R1_5 | PDL1_rMean_std | 0.426 | 0.003 | 0.012 |
| R1_5 | PDL1_area_skewness | -0.375 | 0.010 | 0.021 |
| R1_5 | PDL1_major_skewness | -0.332 | 0.024 | 0.049 |
| R1_5 | PDL1_minor_skewness | -0.440 | 0.002 | 0.006 |
| R1_5 | PDL1_area_kurtosis | -0.332 | 0.024 | 0.049 |
| R1_5 | PDL1_minor_kurtosis | -0.396 | 0.007 | 0.015 |
| R1_5 | PDL1_area_entropy | 0.350 | 0.017 | 0.034 |
| R1_5 | CAIX_area_bin2 | -0.336 | 0.023 | 0.045 |
| R1_5 | CAIX_major_bin1 | -0.333 | 0.024 | 0.048 |
| R1_5 | CD8_area_bin1 | -0.420 | 0.004 | 0.013 |
| R1_5 | CD8_area_bin6 | 0.386 | 0.008 | 0.016 |
| R1_5 | CD8_area_bin7 | 0.334 | 0.023 | 0.034 |
| R1_5 | CD8_major_bin1 | -0.460 | 0.001 | 0.004 |
| R1_5 | CD8_major_bin5 | 0.416 | 0.004 | 0.010 |
| R1_5 | CD8_major_bin7 | 0.406 | 0.005 | 0.011 |
| R1_5 | CD8_minor_bin2 | -0.371 | 0.011 | 0.022 |
| R1_5 | CD8_minor_bin3 | -0.379 | 0.009 | 0.028 |
| R1_5 | CD8_minor_bin7 | 0.396 | 0.006 | 0.016 |
| R1_5 | CD8_minor_bin8 | 0.343 | 0.020 | 0.028 |
| R1_5 | CD8_rMean_bin9 | 0.517 | 0.000 | 0.001 |
| R1_5 | CD8_rMean_bin10 | 0.413 | 0.004 | 0.012 |
| R1_5 | CD8_gMean_bin9 | 0.378 | 0.010 | 0.042 |
| R1_5 | CD8_gMean_bin10 | 0.510 | 0.000 | 0.001 |
| R1_5 | CD8_area_mean | 0.376 | 0.010 | 0.030 |
| R1_5 | CD8_minor_mean | 0.361 | 0.014 | 0.025 |
| R1_5 | CD8_rMean_std | 0.592 | 0.000 | 0.000 |
| R1_5 | CD8_gMean_std | 0.410 | 0.005 | 0.017 |
| R1_5 | CD8_minor_skewness | -0.367 | 0.012 | 0.018 |
| R1_5 | CD8_area_entropy | 0.381 | 0.009 | 0.024 |
| R1_5 | FOXP3_distMean_bin8 | -0.343 | 0.020 | 0.042 |
| R1_5 | CD68_area_bin1 | -0.418 | 0.004 | 0.010 |
| R1_5 | CD68_area_bin2 | -0.404 | 0.005 | 0.015 |
| R1_5 | CD68_major_bin1 | -0.419 | 0.004 | 0.008 |
| R1_5 | CD68_minor_bin2 | -0.383 | 0.009 | 0.037 |
| R1_5 | CD68_minor_bin3 | -0.425 | 0.003 | 0.009 |
| R1_5 | CD68_gMean_bin10 | 0.445 | 0.002 | 0.012 |
| R1_5 | CD68_minor_skewness | -0.399 | 0.006 | 0.016 |
| R1_5 | CD163_area_bin1 | -0.413 | 0.004 | 0.011 |
| R1_5 | CD163_area_bin2 | -0.380 | 0.009 | 0.018 |
| R1_5 | CD163_major_bin1 | -0.494 | 0.000 | 0.001 |
| R1_5 | CD163_minor_bin1 | -0.400 | 0.006 | 0.018 |
| R1_5 | CD163_minor_bin2 | -0.365 | 0.013 | 0.029 |
| R1_5 | CD163_minor_bin3 | -0.411 | 0.005 | 0.010 |
| R1_5 | CD163_rMean_bin10 | 0.369 | 0.012 | 0.023 |
| R1_5 | CD163_gMean_bin10 | 0.354 | 0.016 | 0.036 |
| R1_5 | CD163_area_skewness | -0.426 | 0.003 | 0.006 |
| R1_5 | CD163_major_skewness | -0.362 | 0.014 | 0.020 |
| R1_5 | CD163_minor_skewness | -0.522 | 0.000 | 0.000 |
| R1_5 | CD163_area_kurtosis | -0.364 | 0.013 | 0.020 |
| R1_5 | CD163_major_kurtosis | -0.354 | 0.016 | 0.031 |
| R1_5 | CD163_minor_kurtosis | -0.501 | 0.000 | 0.001 |
| R1_5 | CD66b_minor_bin6 | 0.347 | 0.018 | 0.037 |
| R1_5 | CD66b_minor_skewness | -0.422 | 0.004 | 0.014 |
| R1_5 | CD66b_minor_kurtosis | -0.389 | 0.007 | 0.027 |
| R1_5 | CD45RO_area_bin1 | -0.434 | 0.003 | 0.013 |
| R1_5 | CD45RO_area_bin2 | -0.336 | 0.023 | 0.045 |
| R1_5 | CD45RO_area_bin6 | 0.361 | 0.014 | 0.028 |
| R1_5 | CD45RO_major_bin1 | -0.467 | 0.001 | 0.002 |
| R1_5 | CD45RO_minor_bin2 | -0.328 | 0.026 | 0.046 |
| R1_5 | CD45RO_minor_bin3 | -0.336 | 0.022 | 0.046 |
| R1_5 | CD45RO_minor_bin6 | 0.336 | 0.023 | 0.043 |
| R1_5 | CD45RO_minor_bin7 | 0.355 | 0.016 | 0.035 |
| R1_5 | CD45RO_rMean_bin9 | 0.390 | 0.007 | 0.033 |
| R1_5 | CD45RO_rMean_bin10 | 0.392 | 0.007 | 0.035 |
| R1_5 | CD45RO_gMean_bin10 | 0.435 | 0.003 | 0.009 |
| R1_5 | CD45RO_distMin_bin1 | -0.364 | 0.013 | 0.034 |
| R1_5 | CD45RO_distMin_bin4 | 0.376 | 0.010 | 0.021 |
| R1_5 | CD45RO_area_mean | 0.345 | 0.019 | 0.038 |
| R1_5 | CD45RO_minor_mean | 0.348 | 0.018 | 0.034 |
| R1_5 | CD45RO_rMean_std | 0.430 | 0.003 | 0.010 |
| R1_5 | CD45RO_minor_skewness | -0.356 | 0.015 | 0.027 |
| R1_5 | CD45RO_area_entropy | 0.341 | 0.020 | 0.041 |
| R1_5 | HE_area_bin1 | -0.396 | 0.006 | 0.018 |
| R1_5 | HE_area_bin2 | -0.371 | 0.011 | 0.022 |
| R1_5 | HE_area_bin6 | 0.333 | 0.024 | 0.047 |
| R1_5 | HE_area_bin7 | 0.331 | 0.025 | 0.049 |
| R1_5 | HE_major_bin1 | -0.426 | 0.003 | 0.006 |
| R1_5 | HE_major_bin7 | 0.333 | 0.024 | 0.036 |
| R1_5 | HE_minor_bin2 | -0.335 | 0.023 | 0.040 |
| R1_5 | HE_minor_bin3 | -0.349 | 0.018 | 0.035 |
| R1_5 | HE_minor_bin6 | 0.320 | 0.030 | 0.044 |
| R1_5 | HE_minor_bin7 | 0.378 | 0.010 | 0.019 |
| R1_5 | HE_rMean_bin10 | 0.375 | 0.010 | 0.036 |
| R1_5 | HE_gMean_bin10 | 0.389 | 0.008 | 0.043 |
| R1_5 | HE_distMean_bin1 | -0.349 | 0.017 | 0.035 |
| R1_5 | HE_distMean_bin3 | 0.344 | 0.019 | 0.038 |
| R1_5 | HE_distMean_bin4 | 0.358 | 0.015 | 0.029 |
| R1_5 | HE_distMean_bin8 | 0.330 | 0.025 | 0.050 |
| R1_5 | HE_distMax_bin1 | -0.331 | 0.025 | 0.049 |
| R1_5 | HE_distMax_bin3 | 0.333 | 0.024 | 0.047 |
| R1_5 | HE_distMax_bin9 | 0.362 | 0.013 | 0.043 |
| R1_5 | HE_distMin_bin1 | -0.387 | 0.008 | 0.018 |
| R1_5 | HE_distMin_bin2 | -0.331 | 0.024 | 0.049 |
| R1_5 | HE_distMin_bin5 | 0.374 | 0.011 | 0.022 |
| R1_5 | HE_distMin_bin6 | 0.354 | 0.016 | 0.036 |
| R1_5 | HE_distMin_bin7 | 0.346 | 0.018 | 0.040 |
| R1_5 | HE_area_mean | 0.348 | 0.018 | 0.036 |
| R1_5 | HE_major_mean | 0.334 | 0.023 | 0.035 |
| R1_5 | HE_minor_mean | 0.348 | 0.018 | 0.031 |
| R1_5 | HE_distMean_mean | 0.357 | 0.015 | 0.030 |
| R1_5 | HE_distMax_mean | 0.346 | 0.018 | 0.037 |
| R1_5 | HE_distMin_mean | 0.355 | 0.016 | 0.031 |
| R1_5 | HE_area_skewness | -0.343 | 0.020 | 0.039 |
| R1_5 | HE_minor_skewness | -0.391 | 0.007 | 0.017 |
| R1_5 | HE_major_kurtosis | -0.356 | 0.015 | 0.026 |
| R1_5 | HE_minor_kurtosis | -0.363 | 0.013 | 0.026 |
| R1_5 | HE_area_entropy | 0.349 | 0.017 | 0.035 |
| R1_5 | HE_distMean_entropy | 0.334 | 0.023 | 0.047 |
| R1_5 | HE_distMax_entropy | 0.331 | 0.025 | 0.049 |
| R1_5 | HE_distMin_entropy | 0.338 | 0.022 | 0.043 |
| R1_6 | PDL1_area_bin1 | 0.449 | 0.002 | 0.003 |
| R1_6 | PDL1_area_bin2 | 0.378 | 0.010 | 0.020 |
| R1_6 | PDL1_area_bin5 | -0.340 | 0.021 | 0.042 |
| R1_6 | PDL1_area_bin6 | -0.387 | 0.008 | 0.016 |
| R1_6 | PDL1_major_bin1 | 0.463 | 0.001 | 0.003 |
| R1_6 | PDL1_major_bin7 | -0.345 | 0.019 | 0.039 |
| R1_6 | PDL1_minor_bin1 | 0.332 | 0.024 | 0.029 |
| R1_6 | PDL1_minor_bin2 | 0.373 | 0.011 | 0.021 |
| R1_6 | PDL1_minor_bin3 | 0.362 | 0.013 | 0.034 |
| R1_6 | PDL1_minor_bin7 | -0.381 | 0.009 | 0.018 |
| R1_6 | PDL1_rMean_bin10 | -0.373 | 0.011 | 0.021 |
| R1_6 | PDL1_gMean_bin10 | -0.342 | 0.020 | 0.041 |
| R1_6 | PDL1_distMin_bin1 | 0.391 | 0.007 | 0.015 |
| R1_6 | PDL1_distMin_bin4 | -0.345 | 0.019 | 0.039 |
| R1_6 | PDL1_area_mean | -0.374 | 0.011 | 0.025 |
| R1_6 | PDL1_minor_mean | -0.347 | 0.018 | 0.037 |
| R1_6 | PDL1_rMean_std | -0.400 | 0.006 | 0.012 |
| R1_6 | PDL1_area_skewness | 0.381 | 0.009 | 0.021 |
| R1_6 | PDL1_major_skewness | 0.345 | 0.019 | 0.049 |
| R1_6 | PDL1_minor_skewness | 0.429 | 0.003 | 0.006 |
| R1_6 | PDL1_area_kurtosis | 0.348 | 0.018 | 0.049 |
| R1_6 | PDL1_minor_kurtosis | 0.390 | 0.007 | 0.015 |
| R1_6 | PDL1_area_entropy | -0.366 | 0.012 | 0.034 |
| R1_6 | CAIX_area_bin2 | 0.337 | 0.022 | 0.045 |
| R1_6 | CAIX_major_bin1 | 0.337 | 0.022 | 0.048 |
| R1_6 | CD8_area_bin1 | 0.395 | 0.007 | 0.013 |
| R1_6 | CD8_area_bin6 | -0.389 | 0.007 | 0.016 |
| R1_6 | CD8_area_bin7 | -0.323 | 0.028 | 0.034 |
| R1_6 | CD8_major_bin1 | 0.445 | 0.002 | 0.004 |
| R1_6 | CD8_major_bin5 | -0.409 | 0.005 | 0.010 |
| R1_6 | CD8_major_bin7 | -0.409 | 0.005 | 0.011 |
| R1_6 | CD8_minor_bin2 | 0.356 | 0.015 | 0.023 |
| R1_6 | CD8_minor_bin3 | 0.354 | 0.016 | 0.032 |
| R1_6 | CD8_minor_bin7 | -0.372 | 0.011 | 0.016 |
| R1_6 | CD8_minor_bin8 | -0.334 | 0.023 | 0.028 |
| R1_6 | CD8_rMean_bin9 | -0.475 | 0.001 | 0.003 |
| R1_6 | CD8_rMean_bin10 | -0.399 | 0.006 | 0.012 |
| R1_6 | CD8_gMean_bin9 | -0.357 | 0.015 | 0.042 |
| R1_6 | CD8_gMean_bin10 | -0.501 | 0.000 | 0.001 |
| R1_6 | CD8_area_mean | -0.357 | 0.015 | 0.030 |
| R1_6 | CD8_minor_mean | -0.339 | 0.021 | 0.026 |
| R1_6 | CD8_rMean_std | -0.576 | 0.000 | 0.000 |
| R1_6 | CD8_gMean_std | -0.393 | 0.007 | 0.017 |
| R1_6 | CD8_minor_skewness | 0.349 | 0.017 | 0.021 |
| R1_6 | CD8_area_entropy | -0.367 | 0.012 | 0.024 |
| R1_6 | FOXP3_distMean_bin8 | 0.339 | 0.021 | 0.042 |
| R1_6 | CD68_area_bin1 | 0.407 | 0.005 | 0.010 |
| R1_6 | CD68_area_bin2 | 0.389 | 0.008 | 0.015 |
| R1_6 | CD68_major_bin1 | 0.419 | 0.004 | 0.008 |
| R1_6 | CD68_minor_bin2 | 0.346 | 0.019 | 0.037 |
| R1_6 | CD68_minor_bin3 | 0.411 | 0.005 | 0.009 |
| R1_6 | CD68_gMean_bin10 | -0.389 | 0.008 | 0.023 |
| R1_6 | CD68_minor_skewness | 0.385 | 0.008 | 0.016 |
| R1_6 | CD163_area_bin1 | 0.409 | 0.005 | 0.011 |
| R1_6 | CD163_area_bin2 | 0.391 | 0.007 | 0.018 |
| R1_6 | CD163_major_bin1 | 0.520 | 0.000 | 0.001 |
| R1_6 | CD163_minor_bin1 | 0.382 | 0.009 | 0.018 |
| R1_6 | CD163_minor_bin2 | 0.358 | 0.015 | 0.029 |
| R1_6 | CD163_minor_bin3 | 0.432 | 0.003 | 0.010 |
| R1_6 | CD163_rMean_bin10 | -0.370 | 0.011 | 0.023 |
| R1_6 | CD163_gMean_bin10 | -0.347 | 0.018 | 0.036 |
| R1_6 | CD163_area_skewness | 0.432 | 0.003 | 0.006 |
| R1_6 | CD163_major_skewness | 0.382 | 0.009 | 0.020 |
| R1_6 | CD163_minor_skewness | 0.526 | 0.000 | 0.000 |
| R1_6 | CD163_area_kurtosis | 0.376 | 0.010 | 0.020 |
| R1_6 | CD163_major_kurtosis | 0.377 | 0.010 | 0.030 |
| R1_6 | CD163_minor_kurtosis | 0.500 | 0.000 | 0.001 |
| R1_6 | CD66b_minor_bin6 | -0.356 | 0.015 | 0.037 |
| R1_6 | CD66b_minor_skewness | 0.394 | 0.007 | 0.014 |
| R1_6 | CD66b_minor_kurtosis | 0.361 | 0.014 | 0.027 |
| R1_6 | CD45RO_area_bin1 | 0.408 | 0.005 | 0.013 |
| R1_6 | CD45RO_area_bin2 | 0.340 | 0.021 | 0.045 |
| R1_6 | CD45RO_area_bin6 | -0.361 | 0.014 | 0.028 |
| R1_6 | CD45RO_major_bin1 | 0.463 | 0.001 | 0.002 |
| R1_6 | CD45RO_major_bin7 | -0.330 | 0.025 | 0.050 |
| R1_6 | CD45RO_minor_bin2 | 0.311 | 0.035 | 0.046 |
| R1_6 | CD45RO_minor_bin3 | 0.335 | 0.023 | 0.046 |
| R1_6 | CD45RO_minor_bin6 | -0.316 | 0.032 | 0.043 |
| R1_6 | CD45RO_minor_bin7 | -0.349 | 0.017 | 0.035 |
| R1_6 | CD45RO_rMean_bin9 | -0.351 | 0.017 | 0.033 |
| R1_6 | CD45RO_rMean_bin10 | -0.349 | 0.017 | 0.035 |
| R1_6 | CD45RO_gMean_bin10 | -0.410 | 0.005 | 0.009 |
| R1_6 | CD45RO_distMin_bin1 | 0.350 | 0.017 | 0.034 |
| R1_6 | CD45RO_distMin_bin4 | -0.375 | 0.010 | 0.021 |
| R1_6 | CD45RO_area_mean | -0.346 | 0.018 | 0.038 |
| R1_6 | CD45RO_minor_mean | -0.335 | 0.023 | 0.034 |
| R1_6 | CD45RO_rMean_std | -0.405 | 0.005 | 0.010 |
| R1_6 | CD45RO_minor_skewness | 0.347 | 0.018 | 0.027 |
| R1_6 | CD45RO_area_entropy | -0.347 | 0.018 | 0.041 |
| R1_6 | HE_area_bin1 | 0.381 | 0.009 | 0.018 |
| R1_6 | HE_area_bin2 | 0.377 | 0.010 | 0.022 |
| R1_6 | HE_area_bin6 | -0.347 | 0.018 | 0.047 |
| R1_6 | HE_area_bin7 | -0.340 | 0.021 | 0.049 |
| R1_6 | HE_major_bin1 | 0.429 | 0.003 | 0.006 |
| R1_6 | HE_major_bin7 | -0.360 | 0.014 | 0.028 |
| R1_6 | HE_minor_bin2 | 0.320 | 0.030 | 0.040 |
| R1_6 | HE_minor_bin3 | 0.358 | 0.015 | 0.035 |
| R1_6 | HE_minor_bin6 | -0.309 | 0.037 | 0.044 |
| R1_6 | HE_minor_bin7 | -0.379 | 0.009 | 0.019 |
| R1_6 | HE_rMean_bin10 | -0.348 | 0.018 | 0.036 |
| R1_6 | HE_gMean_bin10 | -0.359 | 0.014 | 0.043 |
| R1_6 | HE_distMean_bin1 | 0.352 | 0.016 | 0.035 |
| R1_6 | HE_distMean_bin3 | -0.351 | 0.017 | 0.038 |
| R1_6 | HE_distMean_bin4 | -0.360 | 0.014 | 0.029 |
| R1_6 | HE_distMean_bin8 | -0.331 | 0.025 | 0.050 |
| R1_6 | HE_distMax_bin1 | 0.336 | 0.022 | 0.049 |
| R1_6 | HE_distMax_bin3 | -0.339 | 0.021 | 0.047 |
| R1_6 | HE_distMax_bin9 | -0.347 | 0.018 | 0.043 |
| R1_6 | HE_distMin_bin1 | 0.381 | 0.009 | 0.018 |
| R1_6 | HE_distMin_bin2 | 0.336 | 0.023 | 0.049 |
| R1_6 | HE_distMin_bin5 | -0.372 | 0.011 | 0.022 |
| R1_6 | HE_distMin_bin6 | -0.348 | 0.018 | 0.036 |
| R1_6 | HE_distMin_bin7 | -0.342 | 0.020 | 0.040 |
| R1_6 | HE_area_mean | -0.358 | 0.014 | 0.036 |
| R1_6 | HE_major_mean | -0.365 | 0.013 | 0.035 |
| R1_6 | HE_minor_mean | -0.340 | 0.021 | 0.031 |
| R1_6 | HE_distMean_mean | -0.358 | 0.015 | 0.030 |
| R1_6 | HE_distMax_mean | -0.349 | 0.017 | 0.037 |
| R1_6 | HE_distMin_mean | -0.356 | 0.015 | 0.031 |
| R1_6 | HE_area_skewness | 0.355 | 0.015 | 0.039 |
| R1_6 | HE_minor_skewness | 0.384 | 0.008 | 0.017 |
| R1_6 | HE_major_kurtosis | 0.366 | 0.012 | 0.026 |
| R1_6 | HE_minor_kurtosis | 0.363 | 0.013 | 0.026 |
| R1_6 | HE_area_entropy | -0.361 | 0.014 | 0.035 |
| R1_6 | HE_major_entropy | -0.342 | 0.020 | 0.040 |
| R1_6 | HE_distMean_entropy | -0.337 | 0.022 | 0.047 |
| R1_6 | HE_distMax_entropy | -0.337 | 0.022 | 0.049 |
| R1_6 | HE_distMin_entropy | -0.339 | 0.021 | 0.043 |
| R1_4 | HE_cluster_mean | -0.362 | 0.013 | 0.046 |
| R1_5 | HE_cluster_mean | -0.338 | 0.021 | 0.046 |
| R1_6 | HE_cluster_mean | 0.335 | 0.023 | 0.046 |

**Supplementary Table S9:** Correlation analysis of radiomic features related to prognosis with nuclear morphology and hypoxia-immune-related biomarkers from H&E and IHC Slides

| Radiomics features | Pathological features | ρ | P value | Calibrated P value |
| --- | --- | --- | --- | --- |
| R2_1 | PDL1_major_bin3 | 0.478 | 0.012 | 0.023 |
| R2_1 | PDL1_major_bin6 | -0.458 | 0.016 | 0.032 |
| R2_1 | PDL1_major_bin7 | -0.459 | 0.016 | 0.032 |
| R2_1 | PDL1_major_bin9 | -0.477 | 0.012 | 0.030 |
| R2_1 | PDL1_major_bin10 | -0.491 | 0.009 | 0.035 |
| R2_1 | PDL1_ratio_bin7 | -0.535 | 0.004 | 0.016 |
| R2_1 | PDL1_major_mean | -0.475 | 0.012 | 0.025 |
| R2_1 | PDL1_major_std | -0.463 | 0.015 | 0.040 |
| R2_1 | PDL1_bMean_std | -0.477 | 0.012 | 0.032 |
| R2_1 | PDL1_major_entropy | -0.462 | 0.015 | 0.030 |
| R2_1 | CD8_major_bin3 | 0.441 | 0.021 | 0.045 |
| R2_1 | CD8_ratio_bin7 | -0.486 | 0.010 | 0.027 |
| R2_1 | CD19_major_bin3 | 0.462 | 0.015 | 0.032 |
| R2_1 | CD19_major_bin6 | -0.439 | 0.022 | 0.044 |
| R2_1 | CD19_major_bin10 | -0.556 | 0.003 | 0.021 |
| R2_1 | CD19_ratio_bin7 | -0.521 | 0.005 | 0.033 |
| R2_1 | CD19_major_entropy | -0.457 | 0.016 | 0.038 |
| R2_1 | FOXP3_ratio_bin2 | 0.481 | 0.011 | 0.028 |
| R2_1 | CD66b_area_bin6 | -0.479 | 0.011 | 0.044 |
| R2_1 | CD66b_major_bin5 | -0.539 | 0.004 | 0.010 |
| R2_1 | CD66b_major_bin6 | -0.463 | 0.015 | 0.030 |
| R2_1 | CD66b_major_bin8 | -0.509 | 0.007 | 0.013 |
| R2_1 | CD66b_minor_std | -0.471 | 0.013 | 0.026 |
| R2_1 | CD66b_area_kurtosis | 0.417 | 0.030 | 0.049 |
| R2_1 | CD66b_area_entropy | -0.448 | 0.019 | 0.038 |
| R2_1 | CD66b_minor_entropy | -0.502 | 0.008 | 0.015 |
| R2_1 | CD45RO_major_bin3 | 0.465 | 0.015 | 0.029 |
| R2_1 | CD45RO_major_bin6 | -0.447 | 0.019 | 0.039 |
| R2_1 | CD45RO_major_bin7 | -0.448 | 0.019 | 0.038 |
| R2_1 | CD45RO_major_bin10 | -0.491 | 0.009 | 0.034 |
| R2_1 | CD45RO_ratio_bin5 | -0.490 | 0.010 | 0.049 |
| R2_1 | CD45RO_ratio_bin8 | -0.523 | 0.005 | 0.026 |
| R2_1 | CD45RO_bMean_std | -0.471 | 0.013 | 0.035 |
| R2_1 | CD68_ch_index | -0.473 | 0.013 | 0.034 |
| R2_2 | PDL1_major_bin3 | -0.466 | 0.014 | 0.023 |
| R2_2 | PDL1_major_bin10 | 0.438 | 0.022 | 0.044 |
| R2_2 | PDL1_ratio_bin7 | 0.562 | 0.002 | 0.016 |
| R2_2 | CD8_ratio_bin7 | 0.508 | 0.007 | 0.027 |
| R2_2 | CD19_major_bin3 | -0.459 | 0.016 | 0.032 |
| R2_2 | CD19_major_bin10 | 0.470 | 0.013 | 0.037 |
| R2_2 | CD19_ratio_bin7 | 0.497 | 0.008 | 0.033 |
| R2_2 | FOXP3_ratio_bin2 | -0.412 | 0.033 | 0.044 |
| R2_2 | CD66b_major_bin5 | 0.477 | 0.012 | 0.019 |
| R2_2 | CD45RO_major_bin3 | -0.420 | 0.029 | 0.047 |
| R2_2 | CD45RO_ratio_bin5 | 0.461 | 0.016 | 0.049 |
| R2_2 | CD45RO_ratio_bin8 | 0.510 | 0.007 | 0.026 |
| R2_2 | CD68_ch_index | 0.456 | 0.017 | 0.034 |
| R2_3 | PDL1_area_bin3 | -0.469 | 0.013 | 0.048 |
| R2_3 | PDL1_area_bin6 | 0.462 | 0.015 | 0.044 |
| R2_3 | PDL1_area_bin7 | 0.491 | 0.009 | 0.033 |
| R2_3 | PDL1_area_bin8 | 0.516 | 0.006 | 0.023 |
| R2_3 | PDL1_area_bin9 | 0.502 | 0.008 | 0.031 |
| R2_3 | PDL1_major_bin2 | -0.534 | 0.004 | 0.012 |
| R2_3 | PDL1_major_bin3 | -0.516 | 0.006 | 0.020 |
| R2_3 | PDL1_major_bin5 | 0.519 | 0.006 | 0.018 |
| R2_3 | PDL1_major_bin6 | 0.545 | 0.003 | 0.009 |
| R2_3 | PDL1_major_bin7 | 0.574 | 0.002 | 0.009 |
| R2_3 | PDL1_major_bin8 | 0.487 | 0.010 | 0.046 |
| R2_3 | PDL1_major_bin9 | 0.520 | 0.005 | 0.022 |
| R2_3 | PDL1_major_bin10 | 0.490 | 0.010 | 0.035 |
| R2_3 | PDL1_minor_bin8 | 0.517 | 0.006 | 0.024 |
| R2_3 | PDL1_minor_bin10 | 0.504 | 0.007 | 0.034 |
| R2_3 | PDL1_major_mean | 0.559 | 0.002 | 0.007 |
| R2_3 | PDL1_area_std | 0.529 | 0.005 | 0.018 |
| R2_3 | PDL1_major_std | 0.502 | 0.008 | 0.036 |
| R2_3 | PDL1_minor_std | 0.503 | 0.007 | 0.033 |
| R2_3 | PDL1_bMean_std | 0.497 | 0.008 | 0.032 |
| R2_3 | PDL1_area_kurtosis | -0.482 | 0.011 | 0.038 |
| R2_3 | PDL1_area_entropy | 0.498 | 0.008 | 0.033 |
| R2_3 | PDL1_major_entropy | 0.563 | 0.002 | 0.010 |
| R2_3 | CD8_major_bin3 | -0.465 | 0.015 | 0.045 |
| R2_3 | CD8_ratio_bin7 | 0.465 | 0.015 | 0.029 |
| R2_3 | CD19_area_bin10 | 0.498 | 0.008 | 0.033 |
| R2_3 | CD19_major_bin2 | -0.488 | 0.010 | 0.031 |
| R2_3 | CD19_major_bin3 | -0.482 | 0.011 | 0.032 |
| R2_3 | CD19_major_bin6 | 0.512 | 0.006 | 0.018 |
| R2_3 | CD19_major_bin10 | 0.468 | 0.014 | 0.037 |
| R2_3 | CD19_major_mean | 0.486 | 0.010 | 0.036 |
| R2_3 | CD19_major_entropy | 0.495 | 0.009 | 0.038 |
| R2_3 | FOXP3_ratio_bin2 | -0.505 | 0.007 | 0.028 |
| R2_3 | FOXP3_rMean_bin7 | -0.451 | 0.018 | 0.048 |
| R2_3 | FOXP3_area_kurtosis | -0.552 | 0.003 | 0.016 |
| R2_3 | CD163_major_skewness | -0.513 | 0.006 | 0.025 |
| R2_3 | CD163_major_kurtosis | -0.513 | 0.006 | 0.025 |
| R2_3 | CD66b_area_bin6 | 0.504 | 0.007 | 0.044 |
| R2_3 | CD66b_major_bin5 | 0.553 | 0.003 | 0.010 |
| R2_3 | CD66b_major_bin6 | 0.529 | 0.005 | 0.030 |
| R2_3 | CD66b_major_bin8 | 0.639 | 0.000 | 0.003 |
| R2_3 | CD66b_minor_bin8 | 0.456 | 0.017 | 0.045 |
| R2_3 | CD66b_minor_bin9 | 0.493 | 0.009 | 0.036 |
| R2_3 | CD66b_minor_bin10 | 0.568 | 0.002 | 0.016 |
| R2_3 | CD66b_minor_std | 0.573 | 0.002 | 0.007 |
| R2_3 | CD66b_area_skewness | -0.455 | 0.017 | 0.045 |
| R2_3 | CD66b_major_skewness | -0.535 | 0.004 | 0.016 |
| R2_3 | CD66b_area_kurtosis | -0.492 | 0.009 | 0.024 |
| R2_3 | CD66b_major_kurtosis | -0.481 | 0.011 | 0.044 |
| R2_3 | CD66b_area_entropy | 0.592 | 0.001 | 0.006 |
| R2_3 | CD66b_minor_entropy | 0.631 | 0.000 | 0.003 |
| R2_3 | CD45RO_area_bin3 | -0.480 | 0.011 | 0.040 |
| R2_3 | CD45RO_area_bin6 | 0.463 | 0.015 | 0.044 |
| R2_3 | CD45RO_area_bin8 | 0.491 | 0.009 | 0.037 |
| R2_3 | CD45RO_major_bin2 | -0.484 | 0.011 | 0.029 |
| R2_3 | CD45RO_major_bin3 | -0.527 | 0.005 | 0.017 |
| R2_3 | CD45RO_major_bin6 | 0.579 | 0.002 | 0.007 |
| R2_3 | CD45RO_major_bin7 | 0.565 | 0.002 | 0.009 |
| R2_3 | CD45RO_major_bin8 | 0.507 | 0.007 | 0.028 |
| R2_3 | CD45RO_major_bin9 | 0.484 | 0.011 | 0.042 |
| R2_3 | CD45RO_major_bin10 | 0.476 | 0.012 | 0.034 |
| R2_3 | CD45RO_ratio_bin5 | 0.437 | 0.023 | 0.049 |
| R2_3 | CD45RO_ratio_bin8 | 0.463 | 0.015 | 0.036 |
| R2_3 | CD45RO_major_mean | 0.504 | 0.007 | 0.030 |
| R2_3 | CD45RO_area_std | 0.491 | 0.009 | 0.037 |
| R2_3 | CD45RO_bMean_std | 0.514 | 0.006 | 0.024 |
| R2_3 | CD45RO_area_kurtosis | -0.488 | 0.010 | 0.039 |
| R2_3 | CD45RO_major_kurtosis | -0.507 | 0.007 | 0.028 |
| R2_3 | CD45RO_major_entropy | 0.527 | 0.005 | 0.019 |
| R2_3 | HE_major_bin6 | 0.493 | 0.009 | 0.024 |
| R2_3 | HE_major_bin7 | 0.482 | 0.011 | 0.043 |
| R2_3 | HE_major_bin8 | 0.481 | 0.011 | 0.044 |
| R2_3 | HE_major_entropy | 0.487 | 0.010 | 0.040 |
| R2_3 | CD68_ch_index | 0.456 | 0.017 | 0.034 |
| R2_4 | PDL1_area_bin3 | -0.470 | 0.013 | 0.048 |
| R2_4 | PDL1_area_bin6 | 0.457 | 0.016 | 0.044 |
| R2_4 | PDL1_area_bin7 | 0.474 | 0.013 | 0.033 |
| R2_4 | PDL1_area_bin8 | 0.529 | 0.005 | 0.023 |
| R2_4 | PDL1_area_bin9 | 0.517 | 0.006 | 0.031 |
| R2_4 | PDL1_major_bin2 | -0.531 | 0.004 | 0.012 |
| R2_4 | PDL1_major_bin3 | -0.503 | 0.007 | 0.020 |
| R2_4 | PDL1_major_bin5 | 0.509 | 0.007 | 0.018 |
| R2_4 | PDL1_major_bin6 | 0.554 | 0.003 | 0.009 |
| R2_4 | PDL1_major_bin7 | 0.563 | 0.002 | 0.009 |
| R2_4 | PDL1_major_bin8 | 0.479 | 0.011 | 0.046 |
| R2_4 | PDL1_major_bin9 | 0.527 | 0.005 | 0.022 |
| R2_4 | PDL1_major_bin10 | 0.472 | 0.013 | 0.035 |
| R2_4 | PDL1_minor_bin8 | 0.514 | 0.006 | 0.024 |
| R2_4 | PDL1_minor_bin10 | 0.496 | 0.009 | 0.034 |
| R2_4 | PDL1_major_mean | 0.555 | 0.003 | 0.007 |
| R2_4 | PDL1_area_std | 0.538 | 0.004 | 0.018 |
| R2_4 | PDL1_major_std | 0.493 | 0.009 | 0.036 |
| R2_4 | PDL1_minor_std | 0.498 | 0.008 | 0.033 |
| R2_4 | PDL1_bMean_std | 0.525 | 0.005 | 0.032 |
| R2_4 | PDL1_area_kurtosis | -0.496 | 0.009 | 0.038 |
| R2_4 | PDL1_area_entropy | 0.498 | 0.008 | 0.033 |
| R2_4 | PDL1_major_entropy | 0.557 | 0.003 | 0.010 |
| R2_4 | CD8_major_bin3 | -0.438 | 0.022 | 0.045 |
| R2_4 | CD8_ratio_bin7 | 0.424 | 0.028 | 0.044 |
| R2_4 | CD19_area_bin10 | 0.504 | 0.007 | 0.033 |
| R2_4 | CD19_major_bin2 | -0.478 | 0.012 | 0.031 |
| R2_4 | CD19_major_bin3 | -0.463 | 0.015 | 0.032 |
| R2_4 | CD19_major_bin6 | 0.507 | 0.007 | 0.018 |
| R2_4 | CD19_major_mean | 0.487 | 0.010 | 0.036 |
| R2_4 | CD19_major_entropy | 0.485 | 0.010 | 0.038 |
| R2_4 | FOXP3_ratio_bin2 | -0.468 | 0.014 | 0.028 |
| R2_4 | FOXP3_area_kurtosis | -0.534 | 0.004 | 0.016 |
| R2_4 | CD163_major_skewness | -0.549 | 0.003 | 0.024 |
| R2_4 | CD163_major_kurtosis | -0.543 | 0.003 | 0.025 |
| R2_4 | CD66b_area_bin6 | 0.457 | 0.017 | 0.044 |
| R2_4 | CD66b_major_bin5 | 0.526 | 0.005 | 0.010 |
| R2_4 | CD66b_major_bin6 | 0.475 | 0.012 | 0.030 |
| R2_4 | CD66b_major_bin8 | 0.576 | 0.002 | 0.007 |
| R2_4 | CD66b_minor_bin10 | 0.491 | 0.009 | 0.037 |
| R2_4 | CD66b_minor_std | 0.517 | 0.006 | 0.015 |
| R2_4 | CD66b_major_skewness | -0.467 | 0.014 | 0.035 |
| R2_4 | CD66b_area_kurtosis | -0.448 | 0.019 | 0.038 |
| R2_4 | CD66b_area_entropy | 0.532 | 0.004 | 0.011 |
| R2_4 | CD66b_minor_entropy | 0.585 | 0.001 | 0.004 |
| R2_4 | CD45RO_area_bin3 | -0.487 | 0.010 | 0.040 |
| R2_4 | CD45RO_area_bin6 | 0.458 | 0.016 | 0.044 |
| R2_4 | CD45RO_area_bin8 | 0.497 | 0.008 | 0.037 |
| R2_4 | CD45RO_major_bin2 | -0.483 | 0.011 | 0.029 |
| R2_4 | CD45RO_major_bin3 | -0.524 | 0.005 | 0.017 |
| R2_4 | CD45RO_major_bin6 | 0.577 | 0.002 | 0.007 |
| R2_4 | CD45RO_major_bin7 | 0.571 | 0.002 | 0.009 |
| R2_4 | CD45RO_major_bin8 | 0.516 | 0.006 | 0.028 |
| R2_4 | CD45RO_major_bin9 | 0.499 | 0.008 | 0.042 |
| R2_4 | CD45RO_major_bin10 | 0.473 | 0.013 | 0.034 |
| R2_4 | CD45RO_ratio_bin5 | 0.419 | 0.029 | 0.049 |
| R2_4 | CD45RO_ratio_bin8 | 0.451 | 0.018 | 0.036 |
| R2_4 | CD45RO_major_mean | 0.516 | 0.006 | 0.030 |
| R2_4 | CD45RO_area_std | 0.505 | 0.007 | 0.037 |
| R2_4 | CD45RO_bMean_std | 0.542 | 0.004 | 0.024 |
| R2_4 | CD45RO_area_kurtosis | -0.493 | 0.009 | 0.039 |
| R2_4 | CD45RO_major_kurtosis | -0.510 | 0.007 | 0.028 |
| R2_4 | CD45RO_major_entropy | 0.534 | 0.004 | 0.019 |
| R2_4 | HE_major_bin6 | 0.500 | 0.008 | 0.024 |
| R2_4 | HE_major_bin7 | 0.502 | 0.008 | 0.043 |
| R2_4 | HE_major_bin8 | 0.493 | 0.009 | 0.044 |
| R2_4 | HE_major_entropy | 0.505 | 0.007 | 0.040 |
| R2_4 | CD68_ch_index | 0.480 | 0.011 | 0.034 |
| R2_5 | CD19_distMax_bin5 | -0.515 | 0.006 | 0.038 |
| R2_5 | CD19_distMin_bin7 | -0.476 | 0.012 | 0.048 |
| R2_6 | CD19_distMean_bin6 | -0.518 | 0.006 | 0.045 |
| R2_6 | CD19_distMax_bin5 | -0.490 | 0.009 | 0.038 |
| R2_6 | CD19_distMin_bin7 | -0.519 | 0.006 | 0.044 |
| R2_6 | FOXP3_ratio_bin2 | 0.419 | 0.030 | 0.044 |
| R2_6 | FOXP3_rMean_bin7 | 0.584 | 0.001 | 0.011 |
| R2_6 | FOXP3_gMean_bin7 | 0.510 | 0.007 | 0.041 |
| R2_6 | FOXP3_gMean_bin8 | 0.499 | 0.008 | 0.040 |
| R2_6 | FOXP3_bMean_bin7 | 0.497 | 0.008 | 0.034 |
| R2_6 | CD66b_rMean_bin3 | -0.518 | 0.006 | 0.045 |
| R2_6 | HE_rMean_bin4 | -0.528 | 0.005 | 0.037 |
| R2_6 | HE_rMean_bin8 | 0.531 | 0.004 | 0.035 |
| R2_7 | PDL1_area_bin3 | -0.452 | 0.018 | 0.048 |
| R2_7 | PDL1_area_bin6 | 0.499 | 0.008 | 0.044 |
| R2_7 | PDL1_area_bin7 | 0.486 | 0.010 | 0.033 |
| R2_7 | PDL1_area_bin8 | 0.467 | 0.014 | 0.037 |
| R2_7 | PDL1_major_bin2 | -0.529 | 0.005 | 0.012 |
| R2_7 | PDL1_major_bin3 | -0.510 | 0.007 | 0.020 |
| R2_7 | PDL1_major_bin5 | 0.607 | 0.001 | 0.006 |
| R2_7 | PDL1_major_bin6 | 0.576 | 0.002 | 0.009 |
| R2_7 | PDL1_major_bin7 | 0.473 | 0.013 | 0.032 |
| R2_7 | PDL1_major_bin9 | 0.464 | 0.015 | 0.030 |
| R2_7 | PDL1_major_mean | 0.566 | 0.002 | 0.007 |
| R2_7 | PDL1_area_kurtosis | -0.466 | 0.014 | 0.038 |
| R2_7 | PDL1_area_entropy | 0.473 | 0.013 | 0.034 |
| R2_7 | PDL1_major_entropy | 0.477 | 0.012 | 0.030 |
| R2_7 | CAIX_area_bin9 | 0.528 | 0.005 | 0.037 |
| R2_7 | CD8_major_bin3 | -0.562 | 0.002 | 0.018 |
| R2_7 | CD8_minor_bin4 | -0.563 | 0.002 | 0.018 |
| R2_7 | CD8_ratio_bin7 | 0.537 | 0.004 | 0.027 |
| R2_7 | CD19_major_bin2 | -0.515 | 0.006 | 0.031 |
| R2_7 | CD19_major_bin3 | -0.435 | 0.023 | 0.037 |
| R2_7 | CD19_major_bin6 | 0.552 | 0.003 | 0.018 |
| R2_7 | CD19_minor_bin4 | -0.516 | 0.006 | 0.047 |
| R2_7 | CD19_ratio_bin1 | -0.546 | 0.003 | 0.026 |
| R2_7 | CD19_major_mean | 0.469 | 0.013 | 0.036 |
| R2_7 | CD19_major_entropy | 0.449 | 0.019 | 0.038 |
| R2_7 | FOXP3_ratio_bin2 | -0.707 | 0.000 | 0.000 |
| R2_7 | FOXP3_rMean_bin5 | -0.536 | 0.004 | 0.031 |
| R2_7 | FOXP3_rMean_bin6 | -0.622 | 0.001 | 0.004 |
| R2_7 | FOXP3_rMean_bin7 | -0.527 | 0.005 | 0.019 |
| R2_7 | FOXP3_gMean_bin7 | -0.485 | 0.010 | 0.041 |
| R2_7 | FOXP3_gMean_bin8 | -0.486 | 0.010 | 0.040 |
| R2_7 | FOXP3_bMean_bin6 | -0.516 | 0.006 | 0.047 |
| R2_7 | FOXP3_bMean_bin7 | -0.511 | 0.006 | 0.034 |
| R2_7 | FOXP3_area_kurtosis | -0.503 | 0.007 | 0.020 |
| R2_7 | CD66b_area_bin8 | 0.557 | 0.003 | 0.020 |
| R2_7 | CD66b_major_bin5 | 0.629 | 0.000 | 0.003 |
| R2_7 | CD66b_major_bin6 | 0.496 | 0.008 | 0.030 |
| R2_7 | CD66b_major_bin8 | 0.545 | 0.003 | 0.009 |
| R2_7 | CD66b_minor_bin8 | 0.543 | 0.003 | 0.024 |
| R2_7 | CD66b_minor_bin9 | 0.521 | 0.005 | 0.036 |
| R2_7 | CD66b_minor_std | 0.578 | 0.002 | 0.007 |
| R2_7 | CD66b_area_skewness | -0.575 | 0.002 | 0.011 |
| R2_7 | CD66b_major_skewness | -0.540 | 0.004 | 0.016 |
| R2_7 | CD66b_area_kurtosis | -0.602 | 0.001 | 0.007 |
| R2_7 | CD66b_major_kurtosis | -0.498 | 0.008 | 0.044 |
| R2_7 | CD66b_area_entropy | 0.582 | 0.001 | 0.006 |
| R2_7 | CD66b_minor_entropy | 0.595 | 0.001 | 0.004 |
| R2_7 | CD45RO_area_bin3 | -0.463 | 0.015 | 0.040 |
| R2_7 | CD45RO_area_bin6 | 0.504 | 0.007 | 0.044 |
| R2_7 | CD45RO_major_bin2 | -0.515 | 0.006 | 0.029 |
| R2_7 | CD45RO_major_bin3 | -0.510 | 0.007 | 0.017 |
| R2_7 | CD45RO_major_bin5 | 0.533 | 0.004 | 0.034 |
| R2_7 | CD45RO_major_bin6 | 0.543 | 0.003 | 0.009 |
| R2_7 | CD45RO_major_bin7 | 0.493 | 0.009 | 0.024 |
| R2_7 | CD45RO_ratio_bin5 | 0.416 | 0.031 | 0.049 |
| R2_7 | CD45RO_major_mean | 0.455 | 0.017 | 0.046 |
| R2_7 | CD45RO_major_entropy | 0.463 | 0.015 | 0.040 |
| R2_7 | HE_major_bin6 | 0.503 | 0.007 | 0.024 |
| R2_8 | PDL1_area_bin1 | -0.529 | 0.005 | 0.037 |
| R2_8 | PDL1_minor_bin1 | -0.571 | 0.002 | 0.015 |
| R2_8 | PDL1_minor_bin2 | -0.639 | 0.000 | 0.003 |
| R2_8 | PDL1_minor_bin3 | -0.518 | 0.006 | 0.045 |
| R2_8 | PDL1_minor_bin6 | 0.552 | 0.003 | 0.023 |
| R2_8 | PDL1_minor_mean | 0.553 | 0.003 | 0.022 |
| R2_8 | PDL1_minor_skewness | -0.546 | 0.003 | 0.026 |
| R2_8 | CAIX_minor_bin4 | -0.521 | 0.005 | 0.043 |
| R2_8 | CD19_minor_bin1 | -0.539 | 0.004 | 0.030 |
| R2_8 | CD19_minor_bin2 | -0.578 | 0.002 | 0.013 |
| R2_8 | CD68_area_bin1 | -0.514 | 0.006 | 0.048 |
| R2_8 | CD68_minor_bin1 | -0.535 | 0.004 | 0.032 |
| R2_8 | CD68_minor_bin2 | -0.597 | 0.001 | 0.008 |
| R2_8 | CD68_minor_bin3 | -0.561 | 0.002 | 0.019 |
| R2_8 | CD68_minor_bin9 | 0.563 | 0.002 | 0.018 |
| R2_8 | CD68_minor_mean | 0.571 | 0.002 | 0.015 |
| R2_8 | CD163_minor_bin1 | -0.567 | 0.002 | 0.017 |
| R2_8 | CD66b_area_bin1 | -0.662 | 0.000 | 0.001 |
| R2_8 | CD66b_area_bin2 | -0.671 | 0.000 | 0.001 |
| R2_8 | CD66b_major_bin1 | -0.576 | 0.002 | 0.013 |
| R2_8 | CD66b_major_bin2 | -0.585 | 0.001 | 0.011 |
| R2_8 | CD66b_minor_bin1 | -0.530 | 0.004 | 0.036 |
| R2_8 | CD66b_minor_bin2 | -0.543 | 0.003 | 0.028 |
| R2_8 | CD66b_minor_bin3 | -0.611 | 0.001 | 0.006 |
| R2_8 | CD66b_minor_bin8 | 0.514 | 0.006 | 0.024 |
| R2_8 | CD66b_area_skewness | -0.554 | 0.003 | 0.011 |
| R2_8 | CD66b_major_skewness | -0.454 | 0.017 | 0.035 |
| R2_8 | CD66b_minor_skewness | -0.572 | 0.002 | 0.015 |
| R2_8 | CD66b_area_kurtosis | -0.551 | 0.003 | 0.012 |
| R2_8 | CD66b_minor_kurtosis | -0.603 | 0.001 | 0.007 |
| R2_8 | CD66b_area_entropy | 0.435 | 0.023 | 0.038 |
| R2_8 | CD45RO_area_bin1 | -0.611 | 0.001 | 0.006 |
| R2_8 | CD45RO_minor_bin1 | -0.673 | 0.000 | 0.001 |
| R2_8 | CD45RO_minor_bin2 | -0.663 | 0.000 | 0.001 |
| R2_8 | CD45RO_minor_bin3 | -0.518 | 0.006 | 0.045 |
| R2_8 | CD45RO_minor_bin5 | 0.545 | 0.003 | 0.026 |
| R2_8 | CD45RO_minor_bin6 | 0.556 | 0.003 | 0.021 |
| R2_8 | CD45RO_minor_bin7 | 0.591 | 0.001 | 0.009 |
| R2_8 | CD45RO_minor_mean | 0.598 | 0.001 | 0.008 |
| R2_8 | CD45RO_minor_skewness | -0.589 | 0.001 | 0.010 |
| R2_8 | HE_area_bin1 | -0.540 | 0.004 | 0.029 |
| R2_8 | HE_minor_bin2 | -0.614 | 0.001 | 0.005 |
| R2_8 | HE_minor_bin7 | 0.530 | 0.004 | 0.036 |
| R2_8 | HE_minor_mean | 0.540 | 0.004 | 0.029 |
| R2_8 | HE_minor_skewness | -0.523 | 0.005 | 0.041 |

**Supplementary Figure S3:** Correlation analysis of radiomic features related to immunotherapy response and prognosis with 12 cell spatial distribution features from IHC Slides


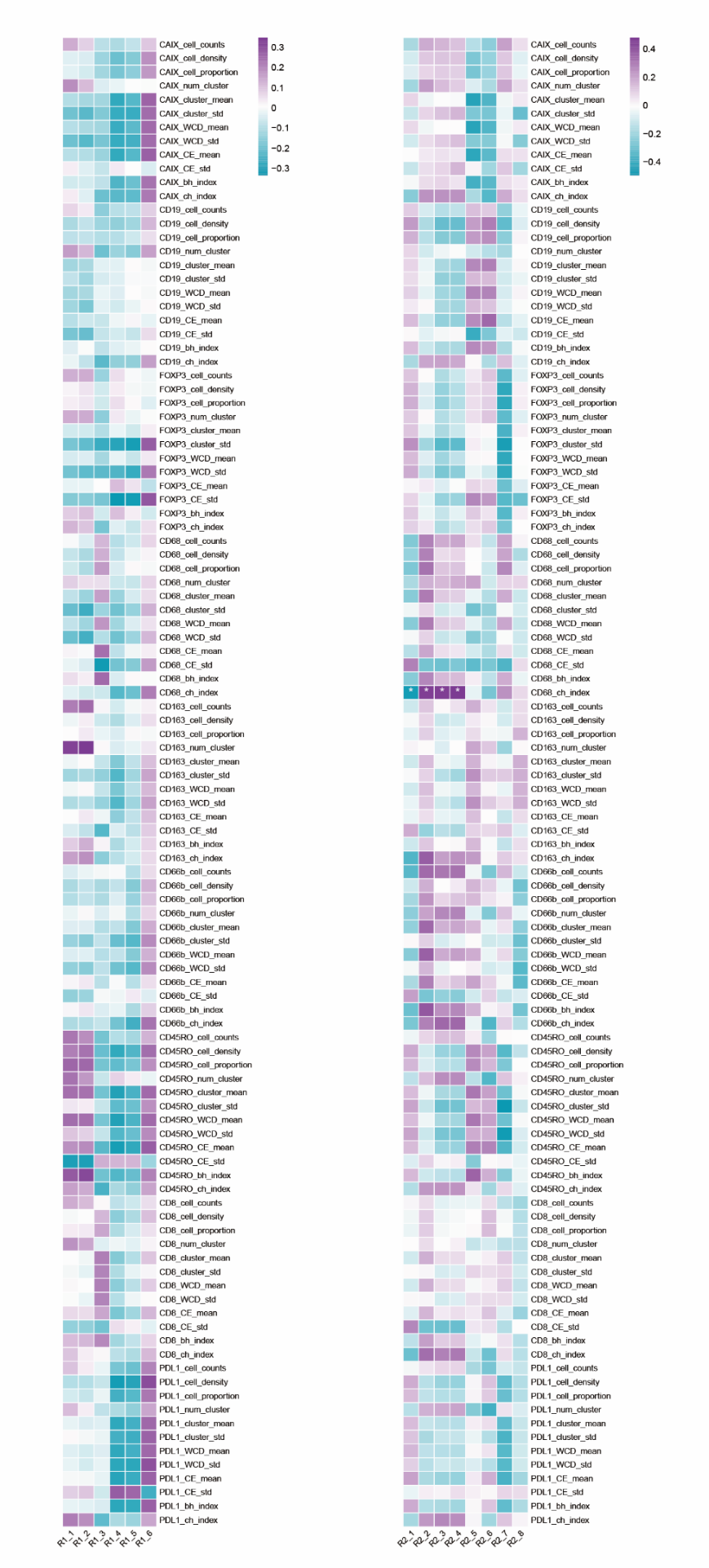


**Supplementary Figure S4**: Patient inclusion and exclusion flowchart.

**
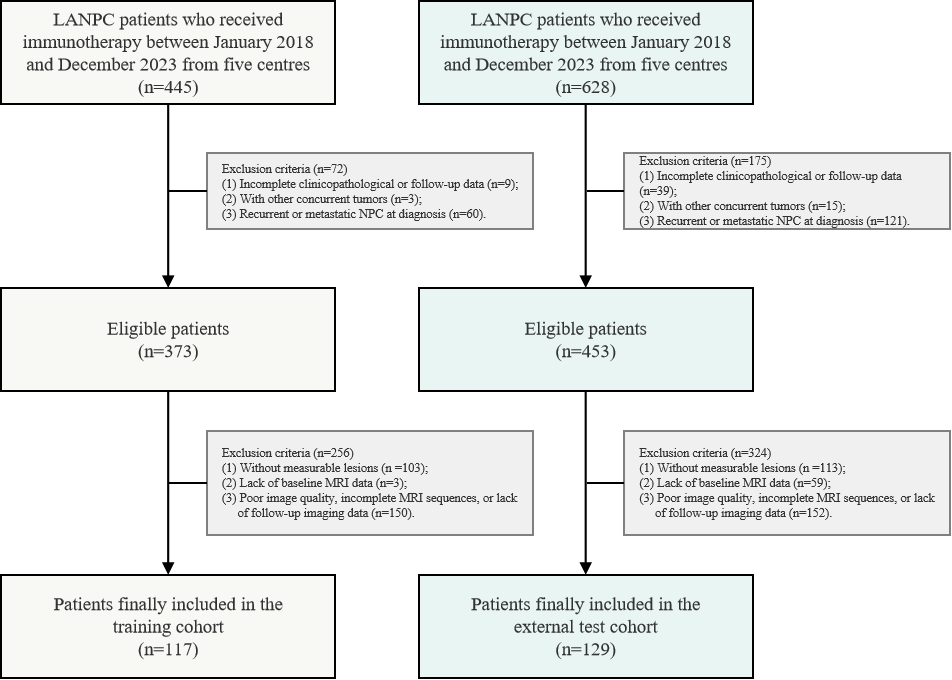
**

**Supplementary Table S10.** MR image acquisition protocol

| **Parameters** | Center 1 | | | | Center 2 | |
| --- | --- | --- | --- | --- | --- | --- |
| **Imaging parameters** | Siemens Skyra | Siemens Magnetom Verio | Siemens Symphony | Siemens PRISMA | Philips  Achieva | GE Discovery |
| **Magnetic Field Strength (T)** | 3.0T | 3.0T | 1.5T | 3.0T | 1.5T | 3.0T |
| **Head coil channel** |  | 24 | 18 | 20 | 16 | 16 |
| **cT1WI** |  |  |  |  |  |  |
| **Repetition Time (ms)** | 515 | 500 | 574 | 725 | 545 | 506 |
| **Echo Time (ms)** | 9.9 | 9.1 | 7.7 | 9.1 | 14.0 | 14.0 |
| **Section thickness (mm)** | 3.0 | 5.0 | 5.0 | 4 | 4.0 | 5.0 |
| **Intersection gap (mm)** |  | 5.5 | 6.5 | 0.4 | 4.0 | 5.0 |
| **Flip Angle (°)** |  | 150 | 90 | 140 | 90 | 110 |
| **Number of Signals Averaged** |  | 1 | 2 | 1 | 1 | 0.7 |
| **Field of View(mm)** | 220×220 | 220 | 230 | 220×206 | 260×250 | 260×250 |
| **Matrix** |  | 324×384 | 512×416 | 320×320 | 252×219 | 256×192 |
| **T1WI** |  |  |  |  |  |  |
| **Repetition Time (ms)** | 400 | 500 | 400 | 580 | 545 | 575 |
| **Echo Time (ms)** | 9.90 | 9.10 | 7.70 | 9.7 | 14.00 | 20.00 |
| **Section thickness (mm)** | 3.0 | 5.0 | 5.0 | 4 | 4.0 | 5.0 |
| **Intersection gap (mm)** |  | 5.5 | 6.0 | 0.4 | 4.0 | 5.0 |
| **Flip Angle (°)** |  | 150 | 90 | 120 | 90 | 110 |
| **Number of Signals Averaged** |  | 1 | 2 | 2 | 1 | 2 |
| **Field of View(mm)** | 220×220 | 220 | 230 | 220×206 | 260×250 | 260×250 |
| **Matrix** |  | 272×320 | 512×448 | 320×320 | 328×220 | 256×192 |
| **T2WI** |  |  |  |  |  |  |
| **Repetition Time (ms)** | 4000 | 4200 | 4000 | 5010 | 3193 | 4000 |
| **Echo Time (ms)** | 78 | 65 | 98 | 83 | 80 | 90 |
| **Inversion Time (ms)** |  |  |  |  |  |  |
| **Section thickness (mm)** | 3.0 | 5.0 | 5.0 | 4 | 5.0 | 5.0 |
| **Intersection gap (mm)** |  | 5.6 | 6.5 | 0.4 | 5.0 | 5.0 |
| **Flip Angle (°)** |  | 150 | 150 | 120 | 90 | 142 |
| **Number of Signals Averaged** |  | 1 | 1 | 2 | 1 | 2 |
| **Field of View(mm)** | 220×220 | 220 | 230 | 220×206 | 260×250 | 260×250 |
| **Matrix** |  | 384×330 | 256×320 | 320×320 | 228×185 | 256×192 |

**Supplementary Table S11.** MR image acquisition protocol

| **Parameters** | Center 3 | | | Center 4 | |
| --- | --- | --- | --- | --- | --- |
| **Imaging parameters** | Philips Primary | Siemens Aera | Siemens Espree | GE Discovery750 | GE Optima MR360 |
| **Magnetic Field Strength (T)** | 3.0T | 1.5T | 1.5T | 3.0T | 1.5T |
| **Head coil channel** | 20 | 16 | 16 | 8 | 8 |
| **cT1WI** |  |  |  |  |  |
| **Repetition Time (ms)** | 570 | 7.24 | 423 | 725 | 539 |
| **Echo Time (ms)** | 12.0 | 2.4 | 4.1 | 3.0 | 3.0 |
| **Section thickness (mm)** | 4.0 | 4.0 | 4.0 | 5.0 | 5.0 |
| **Intersection gap (mm)** | 0.8 | 0.8 | 0.8 | 2.0 | 1.0 |
| **Flip Angle (°)** | 120 | 12 | 70 | 111 | 160 |
| **Number of Signals Averaged** | 2 | 2 | 1 | 1 | 1 |
| **Field of View(mm)** | 220 | 270 | 240 | 220 | 230 |
| **Matrix** | 251×256 | 230×256 | 186×256 | 192×288 | 160×288 |
| **T1WI** |  |  |  |  |  |
| **Repetition Time (ms)** | 520.0 | 510 | 615 | 696 | 564 |
| **Echo Time (ms)** | 12.00 | 8.60 | 11.00 | 3.00 | 3.00 |
| **Section thickness (mm)** | 4.0 | 4.0 | 4.0 | 5.0 | 5.0 |
| **Intersection gap (mm)** | 0.8 | 0.8 | 0.8 | 2.0 | 1.0 |
| **Flip Angle (°)** | 120 | 150 | 150 | 111 | 160 |
| **Number of Signals Averaged** | 1 | 2 | 2 | 2 | 2 |
| **Field of View(mm)** | 230 | 240 | 240 | 220 | 230 |
| **Matrix** | 256×320 | 220×256 | 220×256 | 192×288 | 192×288 |
| **T2WI** |  |  |  |  |  |
| **Repetition Time (ms)** | 4670 | 3700 | 5010 | 5155 | 5465 |
| **Echo Time (ms)** | 89 | 99 | 103 | 12 | 16 |
| **Inversion Time (ms)** | 230 | 160 | 160 | 175 | 150 |
| **Section thickness (mm)** | 4.0 | 4.0 | 4.0 | 5.0 | 5.0 |
| **Intersection gap (mm)** | 0.8 | 0.8 | 0.8 | 2.0 | 1.0 |
| **Flip Angle (°)** | 120 | 150 | 150 | 111 | 160 |
| **Number of Signals Averaged** | 2 | 1 | 1 | 1 | 2 |
| **Field of View(mm)** | 230 | 240 | 240 | 220 | 230 |
| **Matrix** | 288×384 | 230×256 | 259×320 | 224×288 | 192×288 |

**Supplementary Table S12.** MR image acquisition protocol

| **Parameters** | Center 5 and Center 8 | | Center 6 and Center 7 | | |
| --- | --- | --- | --- | --- | --- |
| **Imaging parameters** | GE Signa HDxt | GE Discovery750 | Siemens verio | Philips prisma | Siemens skyra |
| **Magnetic Field Strength (T)** | 1.5T | 3.0T | 3.0T | 3.0T | 3.0T |
| **Head coil channel** | 8 | 24 | 24 | 24 | 24 |
| **cT1WI** |  |  |  |  |  |
| **Repetition Time (ms)** | 200 | 700 | 625 | 581 | 983 |
| **Echo Time (ms)** | 2.0 | 15 | 9 | 12 | 12 |
| **Section thickness (mm)** | 2.4 | 3 | 4 | 4 | 4 |
| **Intersection gap (mm)** | 1.6 | 1 | 1.2 | 0.8 | 0.8 |
| **Flip Angle (°)** | 20 | 110 | 70 | 150 | 160 |
| **Number of Signals Averaged** | 1 | 2 | 1 | 2 | 2 |
| **Field of View(mm)** | 260 | 260 | 180 | 180 | 180 |
| **Matrix** | 320×224 | 288×192 | 212x256 | 346×384 | 224 ×320 |
| **T1WI** |  |  |  |  |  |
| **Repetition Time (ms)** | 650 | 705 | 625 | 581 | 983 |
| **Echo Time (ms)** | 3.26 | 15 | 9 | 12 | 12 |
| **Section thickness (mm)** | 5.0 | 3 | 4 | 4 | 4 |
| **Intersection gap (mm)** | 1.5 | 1 | 1.2 | 0.8 | 0.8 |
| **Flip Angle (°)** | 15 | 111 | 70 | 150 | 160 |
| **Number of Signals Averaged** | 1 | 2 | 1 | 2 | 2 |
| **Field of View(mm)** | 260 | 240 | 180 | 180 | 180 |
| **Matrix** | 320×224 | 256×192 | 212x256 | 346×384 | 224 ×320 |
| **T2WI** |  |  |  |  |  |
| **Repetition Time (ms)** | 4800 | 8877 | 625 | 5180 | 3030 |
| **Echo Time (ms)** | 85 | 80 | 9 | 54 | 74 |
| **Inversion Time (ms)** | 150 | 100 |  |  |  |
| **Section thickness (mm)** | 6.0 | 3 | 4 | 4 | 4 |
| **Intersection gap (mm)** | 1.6 | 1 | 1.2 | 0.8 | 0.8 |
| **Flip Angle (°)** | 90 | 142 | 70 | 150 | 124 |
| **Number of Signals Averaged** | 2 | 1 | 1 | 2 | 2 |
| **Field of View(mm)** | 260 | 260 | 180 | 260 | 200 |
| **Matrix** | 256×224 | 256×192 | 212x256 | 346×384 | 346×384 |

**Supplementary Table S13.** MR image acquisition protocol

| **Parameters** | Center 9 | | Center 10 | |
| --- | --- | --- | --- | --- |
| **Imaging parameters** | GE Signa HDxt | GE Optima MR360 | Siemens MAGNETOM ESSENZA | Siemens skyra |
| **Magnetic Field Strength (T)** | 3.0T | 1.5T | 1.5T | 3.0T |
| **Head coil channel** | 32 | 16 | 16 | 24 |
| **cT1WI** |  |  |  |  |
| **Repetition Time (ms)** | 300 | 380 | 540 | 709 |
| **Echo Time (ms)** | 2.52 | 2.496 | 12 | 10 |
| **Section thickness (mm)** | 5 | 5.0 | 5 | 5 |
| **Intersection gap (mm)** | 6 | 7.5 | 6 | 6 |
| **Flip Angle (°)** | 85 | 80 | 150 | 160 |
| **Number of Signals Averaged** | 1 | 1 | 1 | 1 |
| **Field of View(mm)** | 270 | 230 | 178×260 | 245×280 |
| **Matrix** | 512×512 | 512×512 | 256×256 | 320 ×320 |
| **T1WI** |  |  |  |  |
| **Repetition Time (ms)** | 560 | 560 | 468 | 492 |
| **Echo Time (ms)** | 9.56 | 13.632 | 5 | 11 |
| **Section thickness (mm)** | 5.0 | 5.0 | 4 | 5 |
| **Intersection gap (mm)** | 6 | 7.5 | 6 | 6 |
| **Flip Angle (°)** | 90 | 90 | 150 | 150 |
| **Number of Signals Averaged** | 1 | 1 | 2 | 1 |
| **Field of View(mm)** | 270 | 230 | 178×260 | 245×280 |
| **Matrix** | 512×512 | 512×512 | 256×256 | 256×256 |
| **T2WI** |  |  |  |  |
| **Repetition Time (ms)** | 3860 | 5167 | 7800 | 6020 |
| **Echo Time (ms)** | 98 | 45.396 | 100 | 94 |
| **Inversion Time (ms)** | 0 | 150 |  |  |
| **Section thickness (mm)** | 5 | 5 | 5 | 5 |
| **Intersection gap (mm)** | 6 | 7.5 | 6 | 6 |
| **Flip Angle (°)** | 90 | 90 | 150 | 150 |
| **Number of Signals Averaged** | 1 | 2 | 1 | 1 |
| **Field of View(mm)** | 270 | 230 | 185×260 | 245×280 |
| **Matrix** | 512×512 | 512×512 | 256×256 | 320×320 |

**Supplementary Table S14.** The extracted radiomic features

**Parameters in radiomic feature extraction**

Three types of parameters were used for radiomics feature extraction with an open-source python package (Pyradiomics, Python 3.7.6). The parameters were seen as below:

i) Image type: ‘Original’, ‘LoG’, ‘Wavelet’.

For the derived images, Laplacian of Gaussian (LoG) filter was used for image filtration with ‘sigma’ values of 1, 3, and 5, respectively. Wavelet filter was applied to focus features on the different decomposition and approximation level of the original contoured volumes, and the bin width of which was set as 10.

ii) Image setting: ‘geometryTolerance’, ‘binWidth’, ‘interpolator’, ‘resampledPixelSpacing’.

The geometry tolerance was set as 1.00000e-4. Voxels in each volume were resampled to a unified voxel size of 1×1×1 mm^3^ using ‘sitkBSpline’ while the bin width was set as 25.

iii) Feature Class: ‘shape’, ’firstorder’, ’glcm’, ‘glrlm’, ‘glszm’, ‘gldm’, ‘ngtdm’.

- **Types of radiomic features**

| **Feature group** | **Number** | **Feature name** | **Feature name** |
| --- | --- | --- | --- |
| Shape | 14 | Flatness | Mesh volume |
|  |  | Least axis length | Minor axis length |
|  |  | Major axis length | Sphericity |
|  |  | Maximum 2D diameter (Column) | Surface area |
|  |  | Maximum 2D diameter (Row) | Surface area to volume ratio |
|  |  | Maximum 2D diameter (Slice) | Voxel volume |
|  |  | Maximum 3D Diameter | Elongation |
| First-order | 18 | 10th percentile | Median |
|  |  | 90th percentile | Minimum |
|  |  | Energy | Range |
|  |  | Entropy | Robust mean absolute deviation |
|  |  | Interquartile range | Root mean squared |
|  |  | Kurtosis | Skewness |
|  |  | Maximum | Total Energy |
|  |  | Mean absolute deviation | Uniformity |
|  |  | Mean | Variance |
| GLCM | 24 | Auto correlation | Inverse difference normalized |
|  |  | Cluster prominence | Informational measure of correlation1 |
|  |  | Cluster shade | Informational measure of correlation2 |
|  |  | Cluster tendency | Inverse variance |
|  |  | Contrast | Joint average |
|  |  | Correlation | Joint energy |
|  |  | Difference average | Joint entropy |
|  |  | Difference entropy | Maximal correlation coefficient |
|  |  | Difference variance | Maximum probability |
|  |  | Inverse difference | Sum average |
|  |  | Inverse difference moment | Sum entropy |
|  |  | Inverse difference moment Normalized | Sum squares |
| GLRLM | 16 | Gray level non-uniformity | Run entropy |
|  |  | Gray level non-uniformity Normalized | Run length non-uniformity |
|  |  | Gray level variance | Run length non-uniformity Normalized |
|  |  | High gray level run emphasis | Run percentage |
|  |  | Long run emphasis | Run variance |
|  |  | Long run high gray level Emphasis | Short run emphasis |
|  |  | Long run low gray level Emphasis | Short run high gray level Emphasis |
|  |  | Low gray level run emphasis | Short run low gray level Emphasis |
| GLSZM | 16 | Gray level non-uniformity | Size-zone non-uniformity |
|  |  | Gray level non-uniformity Normalized | Size-zone non-uniformity Normalized |
|  |  | Gray level variance | Small area emphasis |
|  |  | High gray level zone emphasis | Small area high gray level Emphasis |
|  |  | Large area emphasis | Small area low gray level Emphasis |
|  |  | Large area high gray level Emphasis | Zone entropy |
|  |  | Large area low gray level Emphasis | Zone percentage |
|  |  | Low gray level zone emphasis | Zone variance |
| GLDM | 14 | Dependence entropy | Gray level non-uniformity |
|  |  | Dependence non-uniformity | Gray level variance |
|  |  | Dependence non-uniformity Normalized | High gray level Emphasis |
|  |  | Dependence variance | Large dependence Emphasis |
|  |  | Large dependence high gray level Emphasis | Large dependence low gray level Emphasis |
|  |  | Low gray level Emphasis | Small dependence Emphasis |
|  |  | Small dependence high gray level Emphasis | Small dependence low gray level Emphasis |
| NGTDM | 5 | Busyness | Contrast |
|  |  | Coarseness | Strength |
|  |  | Complexity |  |

**Abbreviations:** *GLCM,* gray level co-occurrence matrix; *GLRLM,* gray level run length matrix; *GLSZM,* gray level size zone matrix; *NGTDM,* neighboring gray tone difference matrix.

**Supplementary Table S15.** 150 patient-level nuclear features extracted from H&E WSI

| Ten types of morphological nuclear features, including nuclear area, lengths of the major and minor axes of cell nucleus, the ratio of major axis length to minor axis length (major, minor, and ratio), mean pixel values of nucleus in RGB three channels, and mean, maximum, and minimum distances (distant, distMax, and distMin) to its neighboring nuclei. | Area | area_bin1 |
| --- | --- | --- |
|  |  | area_bin2 |
|  |  | area_bin3 |
|  |  | area_bin4 |
|  |  | area_bin5 |
|  |  | area_bin6 |
|  |  | area_bin7 |
|  |  | area_bin8 |
|  |  | area_bin9 |
|  |  | area_bin10 |
|  | Major | major_bin1 |
|  |  | major_bin2 |
|  |  | major_bin3 |
|  |  | major_bin4 |
|  |  | major_bin5 |
|  |  | major_bin6 |
|  |  | major_bin7 |
|  |  | major_bin8 |
|  |  | major_bin9 |
|  |  | major_bin10 |
|  | Minor | minor_bin1 |
|  |  | minor_bin2 |
|  |  | minor_bin3 |
|  |  | minor_bin4 |
|  |  | minor_bin5 |
|  |  | minor_bin6 |
|  |  | minor_bin7 |
|  |  | minor_bin8 |
|  |  | minor_bin9 |
|  |  | minor_bin10 |
|  | Ratio | ratio_bin1 |
|  |  | ratio_bin2 |
|  |  | ratio_bin3 |
|  |  | ratio_bin4 |
|  |  | ratio_bin5 |
|  |  | ratio_bin6 |
|  |  | ratio_bin7 |
|  |  | ratio_bin8 |
|  |  | ratio_bin9 |
|  |  | ratio_bin10 |
|  | R-Mean | rMean_bin1 |
|  |  | rMean_bin2 |
|  |  | rMean_bin3 |
|  |  | rMean_bin4 |
|  |  | rMean_bin5 |
|  |  | rMean_bin6 |
|  |  | rMean_bin7 |
|  |  | rMean_bin8 |
|  |  | rMean_bin9 |
|  |  | rMean_bin10 |
|  | G-Mean | gMean_bin1 |
|  |  | gMean_bin2 |
|  |  | gMean_bin3 |
|  |  | gMean_bin4 |
|  |  | gMean_bin5 |
|  |  | gMean_bin6 |
|  |  | gMean_bin7 |
|  |  | gMean_bin8 |
|  |  | gMean_bin9 |
|  |  | gMean_bin10 |
|  | B-Mean | bMean_bin1 |
|  |  | bMean_bin2 |
|  |  | bMean_bin3 |
|  |  | bMean_bin4 |
|  |  | bMean_bin5 |
|  |  | bMean_bin6 |
|  |  | bMean_bin7 |
|  |  | bMean_bin8 |
|  |  | bMean_bin9 |
|  |  | bMean_bin10 |
|  | DistMean | distMean_bin1 |
|  |  | distMean_bin2 |
|  |  | distMean_bin3 |
|  |  | distMean_bin4 |
|  |  | distMean_bin5 |
|  |  | distMean_bin6 |
|  |  | distMean_bin7 |
|  |  | distMean_bin8 |
|  |  | distMean_bin9 |
|  |  | distMean_bin10 |
|  | DistMax | distMax_bin1 |
|  |  | distMax_bin2 |
|  |  | distMax_bin3 |
|  |  | distMax_bin4 |
|  |  | distMax_bin5 |
|  |  | distMax_bin6 |
|  |  | distMax_bin7 |
|  |  | distMax_bin8 |
|  |  | distMax_bin9 |
|  |  | distMax_bin10 |
|  | DistMin | distMin_bin1 |
|  |  | distMin_bin2 |
|  |  | distMin_bin3 |
|  |  | distMin_bin4 |
|  |  | distMin_bin5 |
|  |  | distMin_bin6 |
|  |  | distMin_bin7 |
|  |  | distMin_bin8 |
|  |  | distMin_bin9 |
|  |  | distMin_bin10 |
| Five statistic measurements (mean, SD, skewness, kurtosis, and entropy). | Mean | area_mean |
|  |  | major_mean |
|  |  | minor_mean |
|  |  | ratio_mean |
|  |  | rMean_mean |
|  |  | gMean_mean |
|  |  | bMean_mean |
|  |  | distMean_mean |
|  |  | distMax_mean |
|  |  | distMin_mean |
|  | Std | area_std |
|  |  | major_std |
|  |  | minor_std |
|  |  | ratio_std |
|  |  | rMean_std |
|  |  | gMean_std |
|  |  | bMean_std |
|  |  | distMean_std |
|  |  | distMax_std |
|  |  | distMin_std |
|  | Skewness | area_skewness |
|  |  | major_skewness |
|  |  | minor_skewness |
|  |  | ratio_skewness |
|  |  | rMean_skewness |
|  |  | gMean_skewness |
|  |  | bMean_skewness |
|  |  | distMean_skewness |
|  |  | distMax_skewness |
|  |  | distMin_skewness |
|  | kurtosis | area_kurtosis |
|  |  | major_kurtosis |
|  |  | minor_kurtosis |
|  |  | ratio_kurtosis |
|  |  | rMean_kurtosis |
|  |  | gMean_kurtosis |
|  |  | bMean_kurtosis |
|  |  | distMean_kurtosis |
|  |  | distMax_kurtosis |
|  |  | distMin_kurtosis |
|  | entropy | area_entropy |
|  |  | major_entropy |
|  |  | minor_entropy |
|  |  | ratio_entropy |
|  |  | rMean_entropy |
|  |  | gMean_entropy |
|  |  | bMean_entropy |
|  |  | distMean_entropy |
|  |  | distMax_entropy |
|  |  | distMin_entropy |

**Supplementary Table S16.** Description of 10 types of morphological nuclear features and 12 cell spatial distribution features

|  | **Description** | **Sketch map** |
| --- | --- | --- |
| Area | Actual size of the cell nucleus | 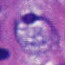 |
| Major axis | The length of the major axis of an ellipse of equal area centered on the cell nucleus | 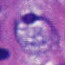 |
| Minor axis | The length of the minor axis of an ellipse of equal area centered on the cell nucleus | 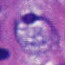 |
| Ratio | The ratio of the length of the major axis to the length of the minor axis of an ellipse of equal area centered on the cell nucleus | 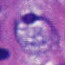 |
| Red channel intensity mean  (R-Mean) | The average intensity of the red (R) channel within the cellular region on the image | 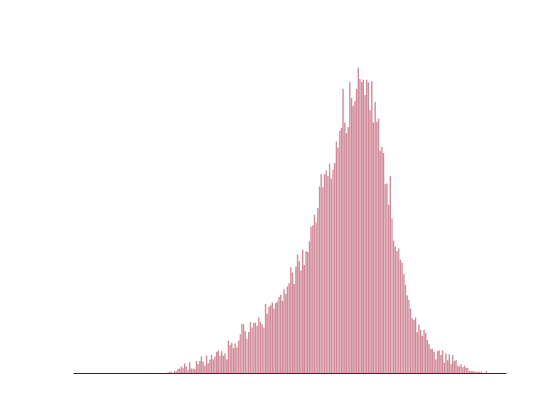 |
| Green channel intensity mean  (G-Mean） | The average intensity of the green (G) channel within the cellular region on the image | 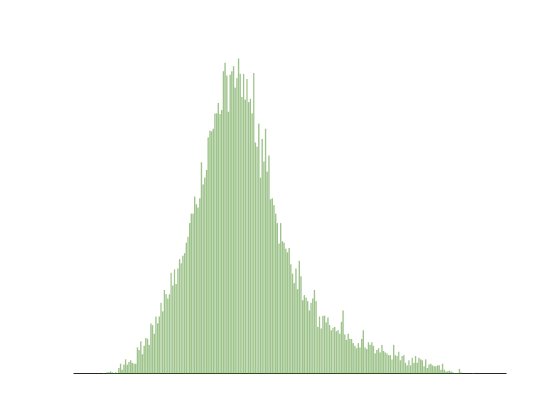 |
| Blue channel intensity mean  (B-Mean) | The average intensity of the blue (B) channel within the cellular region on the image | 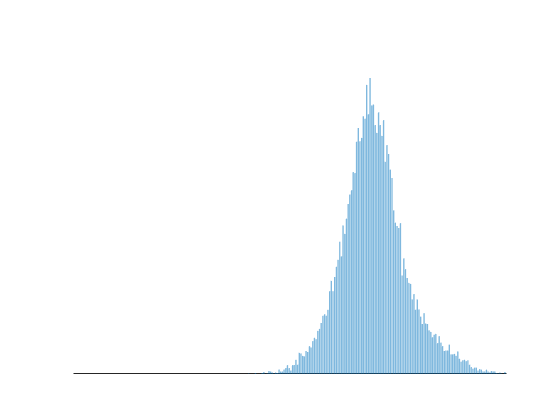 |
| DT graph edge mean  (DistMean) | The average length of all edges connecting cells in a Delaunay triangulation graph constructed with detected cell centers as nodes | 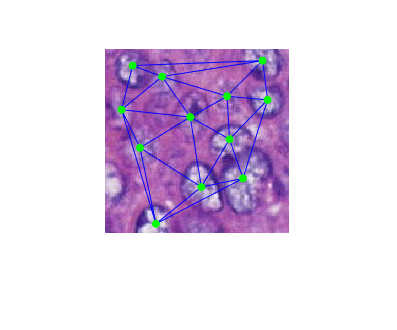 |
| DT graph edge max  (DistMax) | The longest edge length connecting cells in the aforementioned Delaunay triangulation graph | 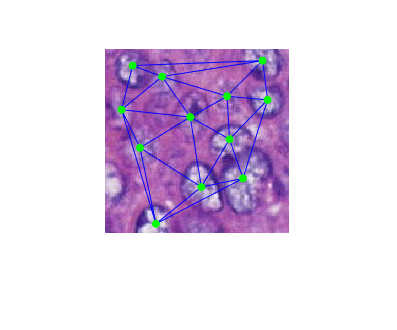 |
| DT graph edge min  (DistMin) | The shortest edge length connecting cells in the aforementioned Delaunay triangulation graph | 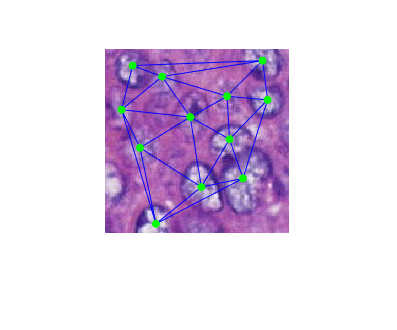 |
| Cell Count & Density | Cell counts: number of cells in the tissue area  Cell densities: dividing the cell counts by the total tissue area | 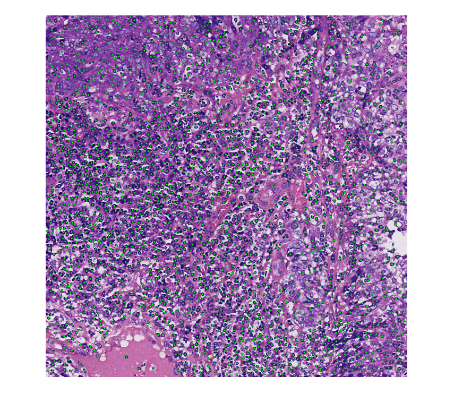 |
| Cell Proportion | Dividing the total cell area by the total tissue area | 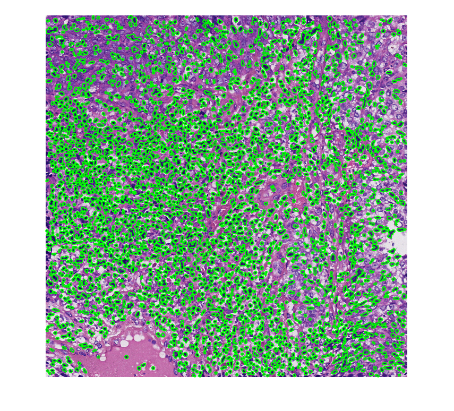 |
| Cell Cluster Features | Cell spatial features based on clustering results by the Birch clustering algorithm | 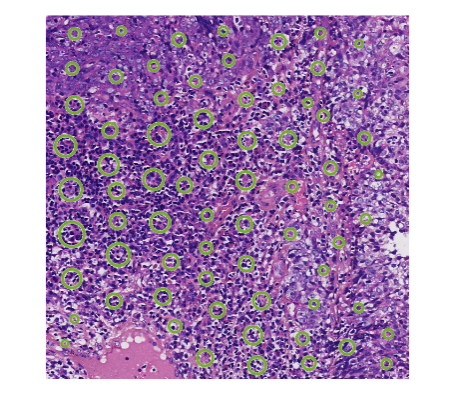 |

**Appendix A1.** Detailed information regarding IHC-staining

1 Experimental equipments and reagents

1.1 Experimental equipments

| **Name** | **Manufacturer** | **Model** |
| --- | --- | --- |
| dehydrator | DIPATH, Italy | Donatello |
| Embedding machine | Wuhan Junjie Electronics Co., LTD | JB-P5 |
| Pathological microtome | Shanghai Leica Instrument Co., LTD | RM2016 |
| Frozen table | Wuhan Junjie Electronics Co., LTD | JB-L5 |
| Tissue machine | Zhejiang Jinhua Kedi Instrument Equipment Co., LTD | KD-P |
| Oven | Shanghai Huitai Instrument Manufacturing Co., LTD | DHG-9140A |
| slide | Servicebio | G6012 |
| Cover glass | Jiangsu Shitai experimental equipment Co., LTD | 10212432C |
| Microwave oven | Galanz microwave electric appliance Co., LTD | P70D20TL-P4 |
| Decolorizing table | Servicebio | DS-2S100 |
| Vortex mixer | Servicebio | MV-100 |
| Palm centrifuge | Servicebio | D1008E |
| Pipette gun | Servicebio |  |
| Tissue pencil | Servicebio | G6100 |
| Microscope | Nikon | E100 |

1.2 Main experimental reagents

| **Name** | **Manufacturer** | **Model** |
| --- | --- | --- |
| Anhydrous ethanol | SCRC | 100092683 |
| Environmentally friendly dewaxing transparent liquid | Servicebio | G1128 |
| xylene | SCRC | 10023418 |
| n-butanol | SCRC | 100052190 |
| Hydrochloric acid | SCRC | 10011028 |
| 20×Citric Acid Antigen Repair Solution (pH 6.0) | Servicebio | G1202 |
| 20×Tris-EDTA Antigen Repair Solution (pH 9.0) | Servicebio | G1203 |
| 20×Tris-EDTA Antigen Repair Solution (pH 8.0) | Servicebio | G1206 |
| PBS buffer | Servicebio | G0002 |
| Universal Tissue Fixative (Neutral) | Servicebio | G1101 |
| 3% hydrogen peroxide disinfectant | Angergech |  |
| Bovine serum albumin BSA | Servicebio | GC305010 |
| Normal rabbit serum (concentrated) | Servicebio | G1209 |
| Hematoxylin dye | Servicebio | G1004 |
| Hematoxylin differentiation solution | Servicebio | G1039 |
| Hematoxylin blue return solution | Servicebio | G1040 |
| Super clean fast drying sealant | Servicebio | G1404-100mL |
| DAB chromogenic reagent for histochemical kit | Servicebio | G1212 |

1.3 Antibody information and repair conditions

| Antigen repair condition | Name of primary antibody | First anti item number | Primary antibody manufacturer | A resistant species | The dilution ratio of one antibody | Name of the corresponding secondary antibody |
| --- | --- | --- | --- | --- | --- | --- |
| EDTA  (pH=9.0)  Medium heat 9min，stop 5min，Medium-Low Heat 7min | CD66b/  CD19/  CD163/  CD8/  FOXP3/  PDL1/  CA-1X/  CD68/  CD45RO | AB300122/  GB11061/  GB113152/  GB114123/  GB112325/  GB14132/  GB121184/  GB14043/  AB23 | abcam/  Servicebio/  Servicebio/  Servicebio/  Servicebio/  Servicebio/  Servicebio/  Servicebio/  abcam | Rab/  Rab/  Rab/  Rab/  Rab/  Mou/  Mou/  Mou/  Mou | 1:20000/  1:500/  1:300/  1:500/  1:600/  1:500/  1:500/  1:100/  1:100 | HRP-conjugated Goat Anti-Rabbit/  HRP-conjugated Goat Anti-Rabbit/  HRP-conjugated Goat Anti-Rabbit/  HRP-conjugated Goat Anti-Rabbit/  HRP-conjugated Goat Anti-Rabbit/  HRP-conjugated Goat Anti-Mouse /  HRP-conjugated Goat Anti-Mouse / HRP-conjugated Goat Anti-Mouse / HRP-conjugated Goat Anti-Mouse / |

Note: The order of adding antibody samples is in accordance with the writing order of antibodies, the number of the second antibody, manufacturer and dilution ratio are shown in the following table.

2 Experimental procedure

2.1 Paraffin sections dewaxing to water: Put the sections into environmentally friendly dewaxing solutionⅠ10 min- Environmentally friendly dewaxing solution Ⅱ10 min- Environmentally friendly dewaxing solution Ⅲ 10 min- anhydrous ethanolⅠ5min- anhydrous ethanol Ⅱ 5 min- anhydrous ethanol Ⅲ 5 min- distilled water in turn.

2.2 Antigen repair: The repair is shown in the table above. During this process, the buffer should be prevented from excessive evaporation and should not be dried. After natural cooling, the slide was placed in PBS (PH7.4) and washed by shaking on the decolorizing shaker for 3 times, 5min each time. (Repair fluid and repair conditions are determined according to the tissue)

2.3 Blocking endogenous peroxidase: The slices were placed in 3% hydrogen peroxide solution, incubated at room temperature away from light for 25 min, and the slides were placed in PBS (PH7.4) and washed three times on a decolorizing shaking table for 5min each time.

2.4 Serum closure: The tissue was uniformly covered with 3%BSA in the tissue chemical circle and closed at room temperature for 30min. (Primary antibody of goat origin is blocked with rabbit serum, other sources are blocked with BSA)

2.5 Add primary antibody: Gently shake off the sealing solution, add PBS to the section in a certain proportion of primary antibody, and the section is placed flat in a wet box at 4°C for overnight incubation.

2.6 Adding secondary antibody: The slide was placed in PBS (PH7.4) and washed by shaking on the decolorizing shaker for 3 times, 5min each time. After the slices were slightly dried, the tissue was covered with the secondary antibody (HRP label) of the corresponding species of the primary antibody, and incubated at room temperature for 50min.

2.7 DAB color development: The slide was placed in PBS (PH7.4) and washed by shaking on the decolorizing table for 3 times, 5min each time. After the sections were slightly dried, the freshly prepared DAB color developing solution was added into the circle. The color developing time was controlled under the microscope. The positive color was brown and yellow, and the sections were rinsed with tap water to terminate the color development.

2.8 Restaining nuclei: hematoxylin restaining for about 3min, washing with tap water, hematoxylin differentiation solution for a few seconds, rinse with tap water, hematoxylin return to blue solution, and rinse with running water.

2.9 Dewatering and sealing: Put the slices into 75% alcohol for 5 min--85% alcohol for 5 min-- anhydrous ethanol for 5 min-- anhydrous ethanol for 5 min--n-butanol for 5 min-- xylene for 5 min to dehydrate and transparent, take the slices out of xylene to dry slightly, and seal the slices with glue.

3 Second antibody

The nucleus of hematoxylin stain is blue, and the positive signal of DAB is brown-yellow.

| Second antibody and TSA name | Model | Manufacturer | Dilution ratio |
| --- | --- | --- | --- |
| CY3 Labeled Goat Anti-Rabbit IgG | GB21303 | Servicebio | 1:300 |
| CY3 tagged Goat anti-mouse IgG | GB21301 | Servicebio | 1:300 |
| CY3 Labeled Goat anti-rat IgG | GB21302 | Servicebio | 1:300 |
| CY3 Labeled Donkey Anti-Goat IgG | GB21404 | Servicebio | 1:300 |
| Cy3-labeled Donkey anti-mouse IgG | GB21401 | Servicebio | 1:300 |
| CY3 Labeled Donkey Anti-Rabbit IgG | GB21403 | Servicebio | 1:300 |
| Alexa Fluor 488 Labeled Goat Anti-Rabbit IgG | GB25303 | Servicebio | 1:400 |
| Alexa Fluor 488 labeled Goat anti-mouse IgG | GB25301 | Servicebio | 1:400 |
| CY5 Labeled Goat anti-mouse IgG | GB27301 | Servicebio | 1:400 |
| CY5 Labeled Goat anti-Rabbit IgG | GB27303 | Servicebio | 1:400 |
| HRP * Polyclonal Rabbit Anti-Goat IgG | GB23204 | Servicebio | 1:200 |
| Goat anti-mouse IgG labeled by HRP | GB23301 | Servicebio | 1:200 |
| HRP * Polyclonal Goat anti-Rat IgG | GB23302 | Servicebio | 1:200 |
| HRP was used to label goat anti-rabbit secondary antibody | GB23303 | Servicebio | 1:200 |
| HRP was used to detect donkey antibody against goat antibody | GB23404 | Servicebio | 1:200 |
| FITC * Donkey Anti-Goat IgG | GB22404 | Servicebio | 1:200 |
| Fitc-labeled Goat anti-rat IgG | GB22302 | Servicebio | 1:200 |
| Fitc-labeled Donkey Anti-Rabbit IgG | GB22403 | Servicebio | 1:200 |
| Fitc-labeled Donkey Anti-Mouse IgG | GB22401 | Servicebio | 1:200 |
| Alexa Fluor 594 Labeled Goat Anti-Rabbit IgG | 111-585-003 | Jackson | 1:400 |
| Alexa Fluor 594 labeled Goat anti-mouse IgG | 115-585-003 | Jackson | 1:400 |
| CY3-Tyramide | G1223 | Servicebio | 1:500 |
| iF488-Tyramide | G1231 | Servicebio | 1:500 |
| iF647-Tyramide | G1232 | Servicebio | 1:400 |
| FITC-Tyramide | G1222 | Servicebio | 1:500 |

**Appendix A2:** Evaluation of PD-L1 Expression Level

The CPS (combined positive score) was determined through the following steps: First, the number of PD-L1 membrane-positive tumor cells and PD-L1 membrane-positive tumor-associated immune cells (including lymphocytes, macrophages, etc.) in each tissue section were counted separately, with tumor-associated immune cells identified according to the 20× rule. Next, the total number of viable tumor cells in the section was assessed, ensuring that at least 100 viable tumor cells were counted. The CPS was calculated using the formula: CPS = (number of PD-L1 membrane - positive tumor cells + number of PD-L1 membrane - positive tumor - associated immune cells) / total number of tumor cells×100. If the calculated value was less than 1, it was reported as CPS <1. To enhance the accuracy and reliability of the results, each NPC (nasopharyngeal carcinoma) tissue section was evaluated twice by two independent pathologists, with the average of the two assessments being taken as the final CPS value. During the evaluations, the pathologists adhered to standardized assessment protocols to ensure consistency and reproducibility. Additionally, all assessments were conducted in a blinded manner with respect to the patients' clinical information to minimize potential biases. Three distinct locations within the nasopharyngeal carcinoma tumor tissue were selected for the coarse needle puncture procedure, and the mean of the CPS scores from these three regions was used as the final CPS score.

**Supplementary Figure S5:** Clustering of TME quantitative features enriched in low- and high-risk patients


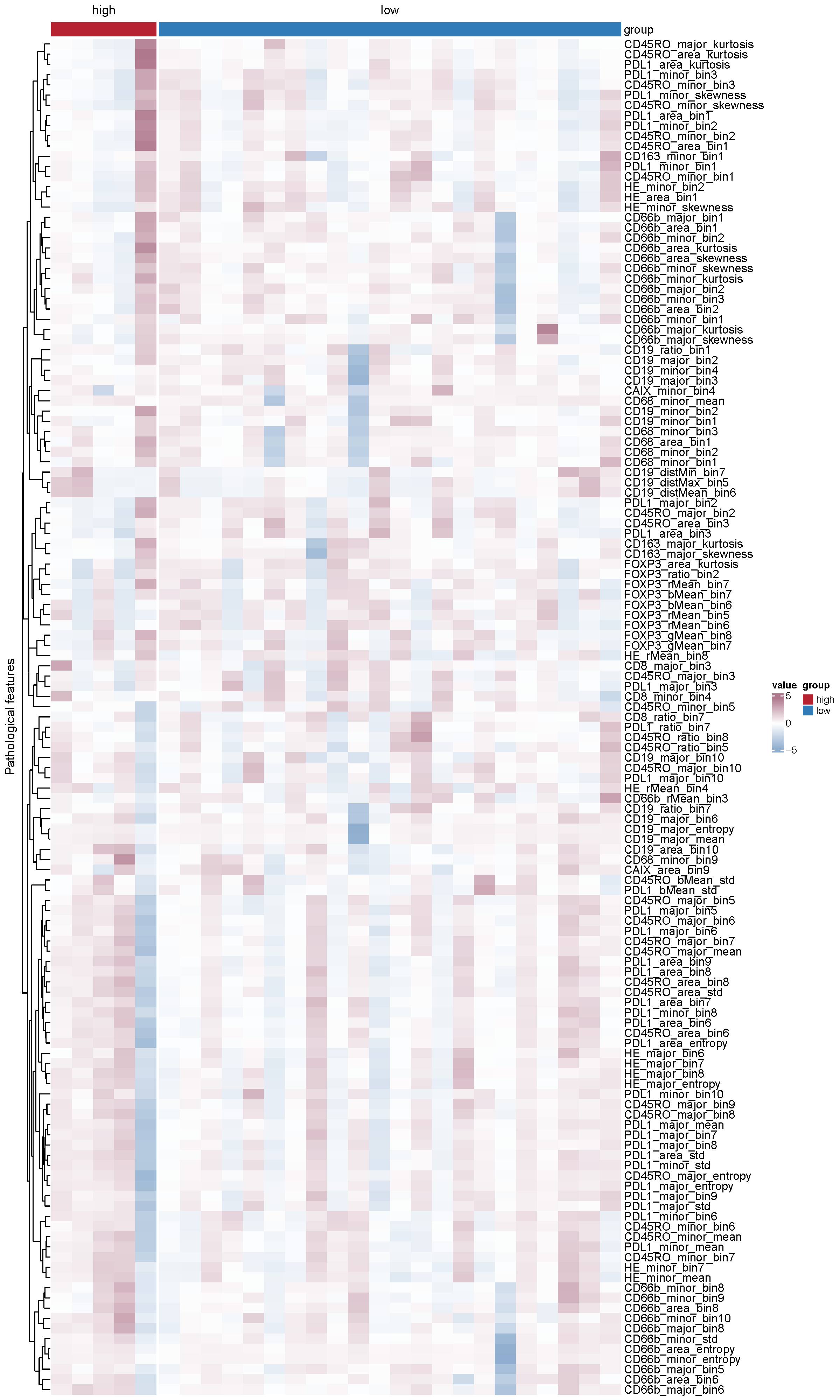


**Supplementary Figure S6:** Correlation Between Radiomic Features with Nuclear Morphological Features from H&E and IHC Slides in the PD-L1 High-Expression Group (CPS >20)


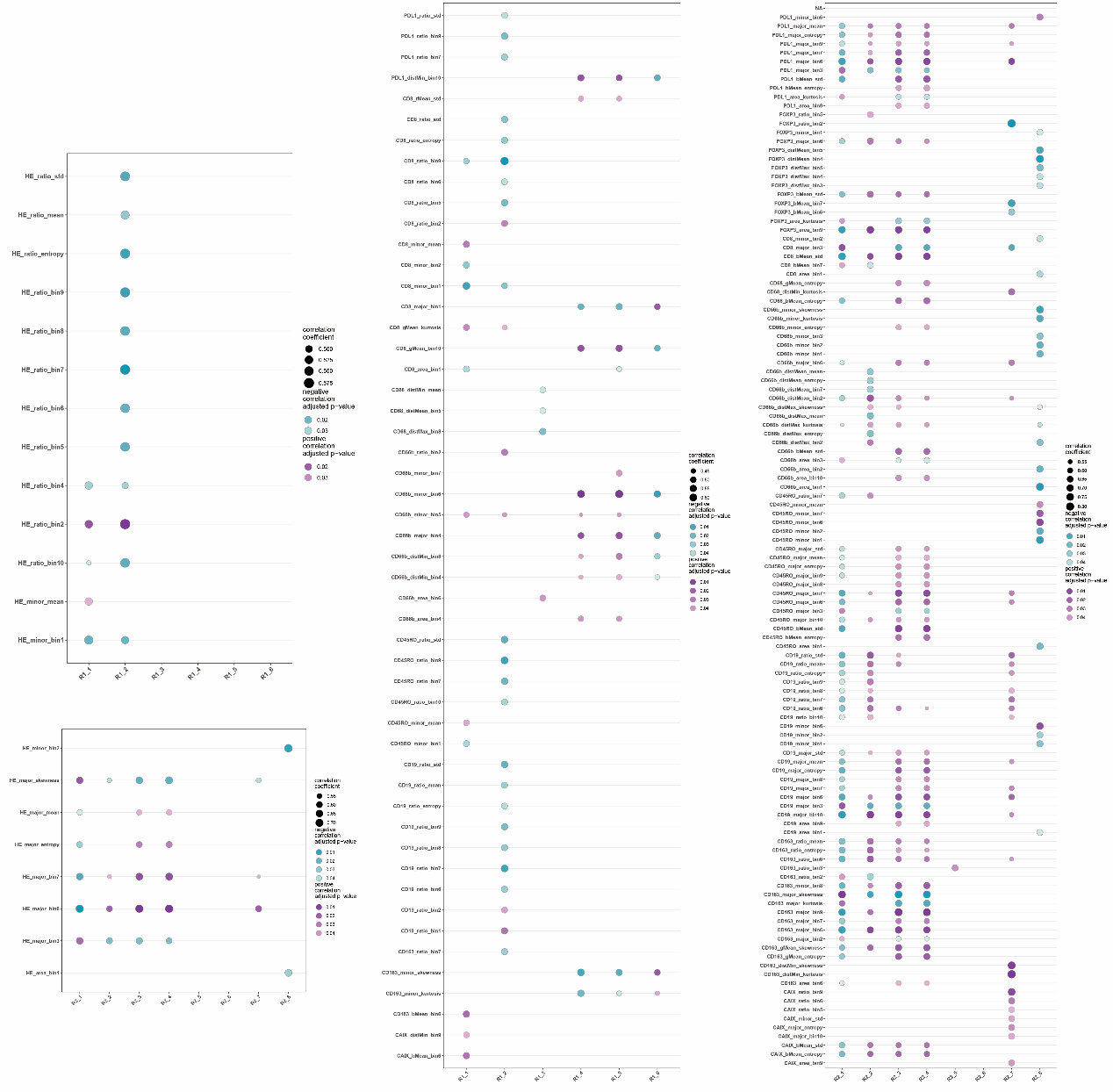

Supplement: Supplementary 1 — Tables S1 to S16 Figs. S1 to S6 Appendices A1 and A2 Supplementary Materials [file research.0749.f1.docx]
